# Propofol and Salvianolic Acid a Synergistically Attenuate LPS‐Induced Myocardial Pyroptosis in Diabetic Mice via the SIRT1/HMGB1 Pathway

**DOI:** 10.1155/mi/6298056

**Published:** 2026-07-07

**Authors:** Anyuan Zhang, Ronghui Han, Kaijia Han, Ting Li, Jianyu Zhu, Yongyan Wang, Jiaqi Zhou, Jiajia Chen, Wangning Shangguan, Stanley Sau Ching Wong, Weiyi Xia, Guanghua Chen, Zhengyuan Xia

**Affiliations:** ^1^ Department of Anesthesiology, The Second Affiliated Hospital and Yuying Children’s Hospital of Wenzhou Medical University, Wenzhou, Zhejiang, China, wmu.edu.cn; ^2^ Department of Anesthesiology, Affiliated Hospital of Guangdong Medical University, Zhanjiang, Guangdong, China, gdmuah.com; ^3^ Faculty of Chinese Medicine, State Key Laboratory of Quality Research in Chinese Medicine, Macau University of Science and Technology, Avenida Wai Long, Taipa, Macao, China, must.edu.mo; ^4^ Department of Anaesthesiology, LKS Faculty of Medicine, The University of Hong Kong, Hong Kong SAR, China, hku.hk; ^5^ Department of Health Technology and Informatics, The Hong Kong Polytechnic University, Hongkong, China, polyu.edu.hk; ^6^ Orthopaedic Center, Affiliated Hospital of Guangdong Medical University, Zhanjiang, Guangdong, China, gdmuah.com

**Keywords:** diabetes, HMGB1, lipopolysaccharide (LPS), propofol (PPF), pyroptosis, salvianolic acid A (SAA), SIRT1

## Abstract

**Background:**

We investigated whether propofol (PPF) combined with salvianolic acid A (SAA) confers synergistic cardioprotection in diabetic sepsis.

**Methods:**

Diabetic‐septic mice and high‐glucose/LPS‐treated cardiomyocytes were treated with PPF and SAA. Cardiac function, reactive oxygen species (ROS), inflammation, and the SIRT1/HMGB1 pathway were assessed.

**Results:**

PPF combined with SAA synergistically attenuated cardiac inflammation, pyroptosis, and dysfunction, accompanied by SIRT1 upregulation and HMGB1 downregulation. Notably, low‐dose coadministration of PPF (12.5 µM) and SAA (12.5 µM) achieved protection comparable to high‐dose PPF (25 µM), significantly reducing ROS and pyroptosis markers. These protective effects were reversed by SIRT1 inhibition or silencing but enhanced by HMGB1 inhibition.

**Conclusion:**

PPF and SAA synergistically inhibit LPS‐induced myocardial pyroptosis under hyperglycemia by activating the SIRT1/HMGB1 pathway. This combination offers a potential strategy to enhance cardioprotection while minimizing anesthetic dosage.

## 1. Introduction

Type 2 diabetes mellitus (T2DM) is a multifactorial chronic metabolic disease that involves not just pancreatic β‐cell disorders but also insulin resistance in various insulin target tissues such as hearts, kidneys, livers, muscles, fat cells, and so on [1, 2]. As an independent form of common cardiovascular diseases, diabetic cardiomyopathy (DCM) possesses a range of specific pathological characteristics and can eventually lead to heart failure [3, 4]. The prognosis of DCM can be dramatically worsened by acute comorbidities like sepsis.

Sepsis remains a major cause of morbidity and mortality worldwide and is generally described as a life‐threatening organ impairment that is featured by the imbalance between the continuous inflammatory response and immune system depression [5]. Cardiac involvement during sepsis frequently occurs, and the manifestation of sepsis in T2DM patients also has become more common [6]. Either sepsis or T2DM will lead to cardiovascular collapse followed by excessive release of reactive oxygen species (ROS), all kinds of inflammatory factors, and initiation of programed cell death such as apoptosis, autophagy, necrosis, and pyroptosis [7, 8].

Substantial evidence has proved that pyroptosis, defined as a new‐type caspase‐1‐dependent programed cell death, is closely related to cardiovascular diseases [9, 10], such as DCM [11]. Pyroptosis has been reported to be stimulated by a range of diseases and activated by inflammatory cytokines, including, but not limited to interleukin (IL)‐1β and IL‐18 [12, 13]. Meanwhile, the increase of ROS could attract the release of nod‐like receptor protein‐3 (NLRP3)‐inflammasome‐mediated pyroptosis that exacerbates myocardial ischemia/reperfusion (I/R) injury in vitro and in vivo [14, 15]. Study revealed that N‐acetylcysteine, a ROS scavenger, inhibited cell pyroptosis induced by lipopolysaccharide (LPS) via the ROS/NLRP3 pathway [16]. Even so, the relationship between pyroptosis and DCM under the condition of sepsis and, in particular, the mechanism of their interplay are largely uninvestigated.

As a member of the sirtuin family, silent information regulator 1 (SIRT1) is a nicotinamide adenosine dinucleotide (NAD)‐dependent deacetylase that can inhibit the overreacting inflammatory responses in sepsis [17] and regulate cardiac functions through various downstream effectors, which are activated by antioxidant signals related to hyperglycemia [18]. Targeting SIRT1 to inhibit pyroptosis has been shown to attenuate myocardial ischemia‐reperfusion injury (MIRI) [19]. In addition, NLRP3 inflammasome‐pyroptosis injury induced by ROS plays a prominent role during MIRI in vivo under or during cardiomyocyte hypoxia–reoxygenation (H/R) in vitro under cytopathologic status, which has also been considered as a potential mechanism to dominate the additional sensitivity of diabetic hearts to sepsis [13, 20]. Furthermore, SIRT1 has also been suggested to be involved in the aforementioned pathologies, in which NLRP3 plays critical roles [21]. Pyroptosis and the rupture of the membrane ultimately ensue as the number of member pores increases during pathological conditions, followed by the release of cellular contents, such as IL‐1α and high‐mobility group box 1 (HMGB1) [22]. The hyperacetylation of HMGB1 is a seminal event prior to its secretion that could be inhibited by SIRT1‐mediated deacetylation [23]. However, whether or not SIRT1 plays a critical role in sepsis‐mediated exacerbation of cardiac injury under hyperglycemic conditions via targeting HMGB1‐mediated pyroptosis is unknown.

ROS is known to play an essential role in various cardiac dysfunctions and sepsis; previous studies by us and others indicated that propofol (PPF) is not only a free‐radical scavenger but also concentration‐dependently attenuates cardiac injuries induced by hyperglycemia and/or ischemia‐reperfusion when used at concentrations higher than those achieved at the typical clinical dosages [24, 25]. On the other hand, Sun et al. [26] offered a unique insight that PPF overdose may initiate cell death of pyroptosis through the NLRP3/Caspase‐1 signal. Fortunately, growing evidence has shown that salvianolic acid A (SAA), a traditional herbal medicine with increasing popularity, may act as a promising drug candidate in cardiovascular disease therapy via its powerful antioxidant activities by inhibiting cell death and inflammation [27]. Crucially, SAA and PPF appear to exert complementary protective effects; while PPF acts as a direct free‐radical scavenger, SAA effectively modulates upstream signaling pathways—such as PKM2‐mediated pyroptosis to inhibit inflammation [27]. Given these distinct but overlapping mechanisms, we hypothesized that a combination of subclinical doses of PPF and SAA could achieve synergistic cardiac protection while mitigating the toxicity associated with high‐dose PPF.

This work was designed to investigate the potential therapeutic effect and underlying mechanism of PPF combined with SAA on LPS‐induced cardiac injuries under hyperglycemia in vitro and in vivo in diabetic mice and to find out whether or not and how NLRP3‐dependent pyroptosis and SIRT1 were involved in the cardioprotective mechanism of PPF combined with SAA.

## 2. Materials and Methods

### 2.1. Animals and Experimental Protocol

Male C57BL/6J wild‐type mice (4–5weeks old, 18 ± 2 g body weight, obtained from the Guangdong Medical Laboratory Animal Center) were used for experiments in vivo. The animal study was approved by the Committee on the Use of Live Animals in Teaching and Research of Guangdong Medical University (Zhanjiang, China), and all experiment protocols and animal handling procedures were conducted according to the recommendations in the principles of animal care. Animals were housed in a controlled environment (12:12‐h light/dark cycle, 22°C ± 2°C, and 60% ± 5% humidity) with ad libitum access to food and water. Following arrival, the mice underwent a 7‐day acclimation period prior to experimental procedures. Mice were randomly divided into 8 groups (*n* = 8–12 per group): NC group (normal control group: received a normal diet and the same volume of citrate buffer injection); T2DM group (treated with a high‐fat diet [HFD, Cat. Number D12492, Research Diets, New Brunswick] for 8 weeks followed by intraperitoneal injection [IP] of streptozotocin [STZ, Sigma] at the dose of 50 mg/kg dissolved in 100 mM citrate buffer (pH 4.5) for 3 consecutive days, subsequently administered with HFD for an additional 8 weeks); NC + LPS group (mice received a normal diet and were exposed to IP injection of LPS [10 mg/kg, Cat. Number L2630, Sigma, Germany]), and T2DM + LPS group (mice were treated with HFD for 8 weeks then received IP injection of STZ at the dose of 50 mg/kg for 3 consecutive days, followed by feeding with HFD for an additional 8 weeks before being exposed to IP injection of LPS at 10 mg/kg [28]. T2DM + LPS + PPF group (PPF [Cat. Number D126608, Sigma, Germany] was injected at a dose of 46 mg/kg/h), and the T2DM + LPS + SAA group (SAA [Cat. Number IS05401, Solarbio, China] was injected at a dose of 10 mg/kg/h). T2DM + LPS + PPF (at a dose of 46 mg/kg/h) + SAA (at a dose of 10 mg/kg/h) group. PPF and SAA were continuously infused via jugular vein catheter using a micro‐infusion pump starting at 2 h before the mice’s sacrifice [29]. T2DM + LPS + PPF + SAA + Ex527 (selective SIRT1 inhibitor, Cat. Number Ab141506, Abcam, UK, group. T2DM + LPS + PPF + SAA + NecroX‐7 (HMGB1 inhibitor, Cat. Number HY‐124750, MCE, Shanghai, China) group.

Blood glucose levels were assessed using tail vein blood 3 days after STZ injection. Mice exhibiting fasting glycemia levels of ≥11.1 mM, along with the presence of elevated blood glucose, polydipsia, and weight loss, were classified as having diabetic conditions [30]. 8 weeks following the STZ injection in the T2DM group, echocardiography (Echo) was performed to confirm the establishment of DCM [31]. For jugular vein catheterization surgery, mice were anesthetized using isoflurane via a precision vaporizer (induction: 3%–4% and maintenance: 1.5%–2% in 100% O_2_). The incision site was infiltrated locally with 0.5% lidocaine prior to the closure. Euthanasia was conducted via controlled CO_2_ asphyxiation (displacement rate: 30%–70% chamber volume/min), followed by cervical dislocation to ensure death, per the institutional protocol. Heart samples were harvested at 6 hr post‐LPS administration and then stored at ‐80°C for further measurements.

### 2.2. Ex527 and NecroX‐7 Treatment

Ex527 (SIRT1 inhibitor) was dissolved in dimethyl sulfoxide and diluted to a final concentration of 2% with normal saline for each medium to minimize any nonspecific or toxic effects; then mice were pretreated for 7 days (0.02 mg/g/day, i.p.) prior to LPS injection [32]. NecroX‐7 was dissolved in phosphate‐buffered saline (PBS, 0.3 mg/kg i.v.) and then injected into mice via tail vein injection at 2‐day intervals for 2 weeks [33].

### 2.3. Echo

Cardiac function was evaluated by Echo at 6 h following the LPS challenge using a high‐resolution imaging system for small animals (Visual Sonics Vevo 2100, Canada). The evaluation of cardiac function involved analyzing parameters on M‐mode images obtained from the parasternal short‐axis view at the level of the papillary muscle. Specifically, the left ventricle internal diameters at end‐diastole and end‐systole were measured, and the percentage of ejection fraction (EF%) and fractional shortening (FS%) were analyzed to assess cardiac function.

### 2.4. Histopathological Analysis

Mice were sacrificed, and their hearts were removed and weighed. The heart was cannulated and retro‐perfused with PBS to remove excess blood. For hematoxylin–eosin (H&E) staining for histopathological analysis, tissues were fixed immediately in 10% neutral buffered formalin and embedded in paraffin. Subsequently, the section with 5 μm was excised from the paraffin blocks and stained with H&E.

### 2.5. Transmission Electron Microscopy (TEM)

For TEM, fresh heart tissue was cut into ≤1 mm^3^ pieces and immediately fixed by immersion in 2.5% glutaraldehyde at 4°C overnight. The fixed samples were washed three times with 0.1 M cacodylate trihydrate buffer and then postfixed with 1% OsO4 for 1 h. After three washes with PB buffer, the samples were dehydrated in the ethanol gradients, incubated with acetone, and then embedded in ethoxyline resin. Ultrathin sections (100 nm) were obtained from a Leica EM UC7 ultramicrotome and then counterstained with uranyl acetate and lead citrate. Samples were subjected to a Tecnai G2 Spirit Biotwin 120 kV Biology Transmission Electron Microscope for ultrastructural analysis, and the mitochondrial number, size, and cristae were assessed with ImageJ.

### 2.6. Cell Culture

The H9c2(2‐1) cell line (CL‐0089, RRID: CVCL_0286) was purchased from Wuhan Pricella Biotechnology Co., Ltd. (Wuhan, China) in 2022. This cell line is a well‐characterized rat (*Rattus norvegicus*) cardiomyoblast cell line derived from the female embryonic ventricular tissue, commonly used for studying cardiac cell biology and pharmacology. The cells were banked and cultured to the 7th generation and then frozen and provided. They were cultured in Dulbecco’s modified Eagle medium supplemented with 10% fetal bovine serum and 1% penicillin/streptomycin at 37°C and 5% CO2. The cell line was authenticated by short tandem repeat profiling, with a 98% match to the reference profile. The authentication certificate was provided by Wuhan Pricella Biotechnology Co., Ltd. The cell line was not reported as misidentified or contaminated in the ICLAC database. Mycoplasma testing was not performed for the described experiments. The cell line was obtained from a commercial supplier with established quality control, and no morphological signs of contamination were observed during the cell culture. This is unlikely to affect the study conclusions as the measured endpoints are robust to potential contamination effects in this experimental context.

### 2.7. Drug Intervention

Cells cultured in a normal glucose (5.6 mM) medium were regarded as the control group (NC). For high glucose (HG) group treatment: when cells reached 50% density, their medium was refreshed with HG medium (35 mM) for 48 h [34]. Then, cells under HG treatment condition were incubated with LPS (10 μg/ml) [35] for 2 h (h) (HG + LPS‐2 h), 4 h (HG + LPS‐4 h), and 6 h (HG + LPS‐6 h) to evaluate the appropriate duration in the following experiments. PPF was categorized into different concentrations ranging from 12.5, 25, 50–100 μM. Similarly, SAA was categorized into different concentrations ranging from 6.25, 12.5, 25, 50–100 μM. The group with coadministration of PPF and SAA invention was additionally annotated as P12.5 + S12.5. The group with NecroX‐7 treatment was added NecroX‐7 at the concentration of 40 μM [36].

### 2.8. SIRT1‐Specific siRNA Transfection

Based on the manufacturer’s manual, specifically designed and synthetic siRNA (50 nmol/L) of SIRT1 (GenePharm, Shanghai, China) was transfected into cells with the recommended agent (Lipofectamine 3000, Invitrogen) to silence SIRT1. The sequences of SIRT1‐siRNA are listed as follows:

SIRT1‐si‐1 : 5’‐CCAAGCAGCUAAGAGUAAUTT‐3’
SIRT1‐si‐2 : 5’‐GCAACUAUACCCAGAACAUTT‐3’
SIRT1‐si‐3 : 5’‐GCUGAUGAACCGCUUGCUATT‐3’

After transfection for 6 h, H9c2 cells were refreshed with hyperglycemia (35 mM) cell culture medium for 48 h and were assigned to continue the experiments. The group with the siRNA transfection invention was additionally annotated as siRNA‐1, siRNA‐2, and siRNA‐3.

### 2.9. Cytotoxicity Analysis

The cell viability was detected by CCK8 (Cat. Number CK04‐100, Dojindo, Japan) and LDH Release Assay Kit (Cat. Number 11644793001, Roche, Switzerland). All cells and cell culture supernatant were processed according to the procedures of the relevant manufacturers. The optical density value of cells and cell culture supernatant was observed at a suitable wavelength (490 nm/450 nm) through a microplate reader, which indirectly reflects the magnitude of viability or damage in cells.

### 2.10. Measurement of ROS

To evaluate the degree of cell injury caused by LPS and HG processing, dihydroethidium (DHE) (Cat. Number S0064S, Beyotime, China), and 2,7‐dichlorodihydrofluorescein diacetate (DCFH‐DA) ROS assay kit (Cat. Number S0033S, Beyotime, China) were used for the detection of intracellular ROS as described. The results of the DCFH‐DA index were measured by a flow cytometer. The results of the ROS level in DHE were detected by a fluorescence microscope.

### 2.11. Biochemical Analysis

Blood was collected as a terminal, nonsurvival procedure immediately following the final ultrasound session. Mice were under deep isoflurane anesthesia (maintenance, 2.5%–3.0%) to ensure unconsciousness and eliminate pain perception. Approximately 1.0–1.2 mL (maximum obtainable volume per mouse) was used. Blood was collected via orbital sinus exsanguination following enucleation. To maximize serum yield, supplemental cardiac massage was performed via gentle thoracic compression (a terminal procedure to facilitate complete blood withdrawal). Blood was transferred to serum separator tubes, allowed to clot at room temperature for 30 min, centrifuged at 3000 rpm for 15 min (4°C), and the serum supernatant was harvested and stored at −80°C until analysis. The levels of aspartate transaminase (AST) and creatine phosphokinase‐MB (CK‐MB) (Donglin Biotechnology, China) in cardiac serum were measured by a chemistry analyzer (HITACHI, Japan).

### 2.12. Inflammatory Cytokines Detection

The levels of inflammatory cytokines in both serum and cell supernatant were evaluated by enzyme‐linked immunosorbent assays, including IL‐6 (Cat. Number JL20896), tumor necrosis factor‐α (TNF‐α, Cat. Number JL13202), IL‐18 (Cat. Number JL20882), and IL‐1β (Cat. Number JL20884), following the manufacturer’s protocols (Jianglai, China). The results were obtained using spectrometry on a 96‐plate reader.

### 2.13. Detection of Total‐Superoxide Dismutase (T‐SOD) and Malondialdehyde (MDA)

In order to assess the extent of myocardial damage, the T‐SOD assay kit (Cat. Number S0101S, Beyotime, China) and MDA assay kit (Cat. Number S0131S, Beyotime, China) was employed to measure antioxidant and lipid peroxidation levels as described [37]. The protein content of cells/tissue in all experimental groups, along with the corresponding reagents in T‐SOD/MDA assay kits. The obtained values were standardized to total protein content, which was determined via a bicinchoninic acid protein assay kit and then added to the aforementioned kits following the manufacturer’s protocols. A microplate reader was used to measure each group’s absorbance at the 450/532 nm wavelength.

### 2.14. Western Blotting Analysis

Cells/tissues from each group were collected and washed twice and then harvested gently with precooled PBS, which then was removed by centrifugation at 200 *g* for 3 min at 4°C. The sedimentation of cells/cardiac digests was lysed with radio immunoprecipitation assay buffer containing protease and phosphatase inhibitor cocktails in the ice bath for 30 min after ultrasonic cracking. Then, soluble protein samples were collected by centrifugation at 12,000 *g* for 10 min at 4°C. Western blot analysis was performed as described [24], using antibodies including SIRT1 (1:1000, Cat. Number 9475, observed MW: 120 kDa, CST, USA); NLRP3 (1:1000, Cat. Number ab263899, observed MW: 110 kDa, Abcam, UK); ASC (1:1000, Cat. Number DF6304, observed MW: 22 kDa, Affinity Biosciences, China); Caspase‐1 (1:1000, Cat. Number 81482‐1‐RR, observed MW: 45 kDa, Proteintech, China); GSDMD (1:1000, Cat. Number ab209845, observed MW: 53 kDa and 32 kDa, Abcam, UK); IL‐18 (1:1000, Cat. Number 33710‐1‐AP, observed MW: 22 kDa, Proteintech, China; IL‐1β (1:1000, Cat. Number 26048‐1‐AP, observed MW: 17kDa, Proteintech, China; and HMGB1 (1:1000, Cat. Number 10829‐1‐AP, observed MW: 29kDa, Proteintech, China). The catalog number for the ladder used is as follows: ladder: Genstar, Catalog Number M221. Data are presented as percentage change relative to GAPDH (1:5000, Cat. Number 10494‐1‐AP, observed MW: 36 kDa, Proteintech, China), serving as a reference while the protein bands were detected using a scan‐imaging instrument. Data are presented as a percent change relative to GAPDH used as the reference.

### 2.15. Statistical Analysis

All data are expressed as the mean ± standard deviation (SD). Statistical analyses were performed using GraphPad Prism (version 10.0, GraphPad Software). Comparisons between two groups were analyzed using an unpaired *t*‐test, and differences among multiple groups were evaluated by one‐way analysis of variance (ANOVA) followed by Tukey’s post‐hoc test. A value of *p*  < 0.05 was considered statistically significant. Statistical significance markers ( ^∗^,  ∗^∗^, and  ∗∗^∗^) in the figures were generated automatically by GraphPad Prism based on calculated *p*‐values, where  ^∗^ *p*  < 0.05,  ^∗∗^ *p*  < 0.002, and  ^∗∗∗^ *p*  < 0.001.

To ensure the validity of our results and adhere to the 3R principles (replacement, reduction, and refinement), the sample size for each experiment was determined using a priori power analysis (G ^∗^Power 3.1.9.7). Based on the effect size derived from our preliminary data, we calculated the minimum number of biological replicates required to achieve a power (1−β) of 0.80 at a significance level of *α* = 0.05. All experiments were performed in at least three independent biological replicates to ensure reproducibility.

## 3. Results

### 3.1. Diabetes Exacerbated LPS‐Induced Cardiac Injury in Mice

**Figure 1:**
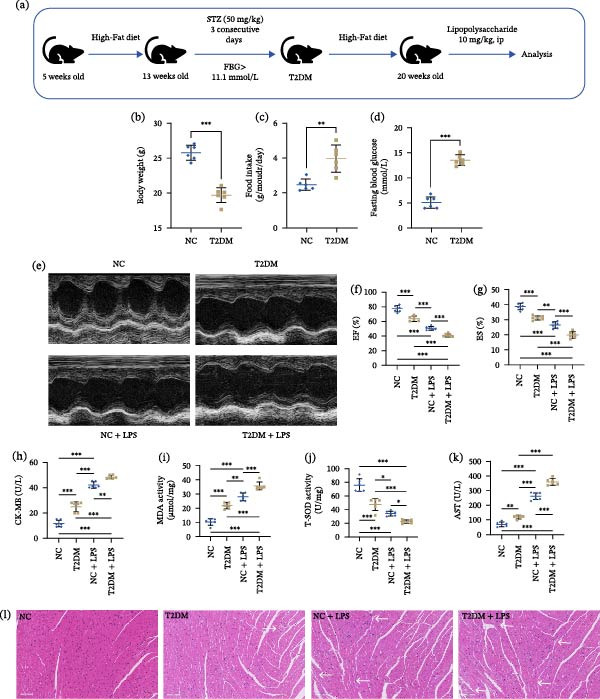
Diabetes aggravated the LPS‐induced cardiac dysfunction in HFD‐STZ‐induced type 2 diabetic mice. (a) Schematic illustrating the experimental design used for model establishment with LPS treatment. (b) Body weight. (c) Food intake. (d) Level of fasting blood glucose. (e) The representative images of the echocardiogram. (f) Ejection fraction (EF%). (g) Fractional shortening (FS%). (h) Creatine kinase‐MB (CK‐MB). (i) Malondialdehyde (MDA). (j) Total‐superoxide dismutase (T‐SOD). (k) Aspartate aminotransferase (AST). (l) Representative histological images from H&E staining heart sections (200×, scale bar: 100 μm); arrows indicate characteristic changes. Data are expressed as mean ± SD,  ^∗^ *p*  < 0.05,  ^∗∗^ *p*  < 0.002,  ^∗∗∗^ *p*  < 0.001, and *n* = 6 per group.

In this study, we established a T2DM‐sepsis model in C57BL/6J mice by intraperitoneally injecting LPS at 10 mg/kg (Figure 1a). The injection of STZ into HFD‐fed mice significantly induced hyperglycemia. There existed a reduction of body weight and an increase of food intake, compared to the NC group (Figure 1b–d). As shown, lasting HG or LPS led to a decline of heart function (including EF% and FS%, Figure 1e–g) and augmentations of cardiac injury indicators CK‐MB and AST (Figure 1h–k) and lipid peroxidation product MDA (Figure 1i) but a reduction in the antioxidant enzyme T‐SOD (Figure 1j), and all of these changes were further exaggerated in the T2DM + LPS group. Furthermore, the histopathological analysis revealed a heightened presence of inflammatory cell infiltration in the T2DM + LPS group compared to both NC + LPS and T2DM groups (Figure 1l). These results indicate that diabetes significantly aggravated the LPS‐induced cardiac dysfunction in HFD/STZ‐induced T2DM mice.

### 3.2. Pyroptosis Was Activated and Exacerbated After LPS Stimulation in Diabetic Mice

**Figure 2:**
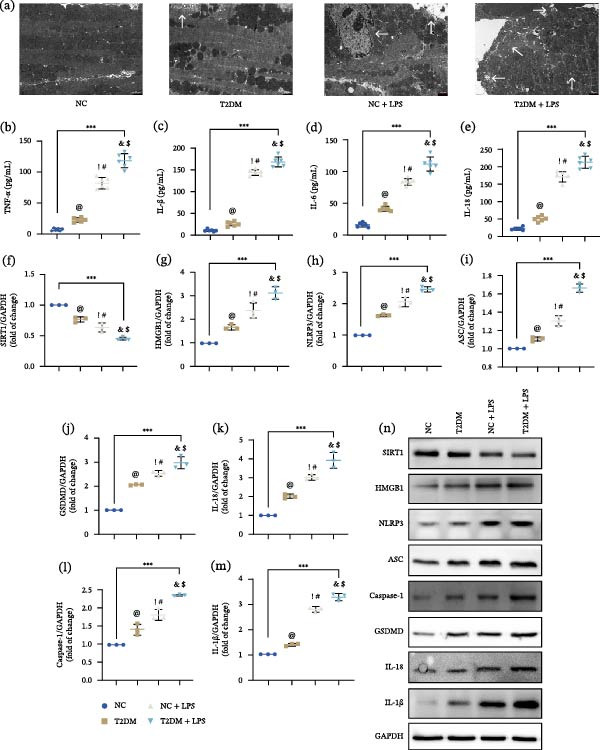
Pyroptosis in hearts was activated and exacerbated after LPS stimulation under diabetes. (a) The results of transmission electron microscopy (TEM, scale bar: 10 μm). Arrows indicate characteristic structural changes, including mitochondrial swelling and cristae disappearance. (b) Tumor necrosis factor (TNF)‐α. (c) Interleukin (IL)‐1β. (d) IL‐6. (e) IL‐18. (f) SIRT1 protein expression. (g) HMGB1 protein expression. (h) NLRP3 protein expression. (i) ASC protein expression. (j) GSDMD protein expression. (k) IL‐18 protein expression. (l) Caspase‐1 protein expression. (m) IL‐1β protein expression. (n) Representative images of western blots. Data are expressed as the mean ± SD, *n* = 6 per group (a–e) and *n* = 3 per group (f–n). ^@^ *p*  < 0.05 vs. NC group, ^!^ *p*  < 0.05 vs. T2DM group, ^#^ *p*  < 0.05 vs. NC group, ^$^ *p*  < 0.05 vs. T2DM group, ^&^ *p*  < 0.05 vs. NC + LPS group, and  ^∗∗∗^ *p*  < 0.001. The grouped gel/blot bands were cropped from the same gel and are clearly delineated in the Supporting Information file.

Previous studies showed that severe inflammation and pyroptosis were activated by LPS in cardiac tissue as a consequence of either activated NLRP3 inflammasome or SIRT1‐related signals [21]. To further explore the potential mechanism whereby LPS aggravates DCM, we analyzed the levels of proinflammatory cytokines and NLRP3 inflammasome‐associated proteins in serum and photographed the myocardial tissue by TEM. The results of TEM showed that the cytoplasm of cardiac H9c2 cells disintegrates to form bubbles, the endoplasmic reticulum ruptures, and condensed matter appears at the center of the cells in both the T2DM and NC + LPS groups, which was more significant in the T2DM + LPS group (Figure 2a). Moreover, the levels of serum inflammatory cytokines, including IL‐18, IL‐6, TNF‐α, and IL‐1β, in the T2DM + LPS group was higher than those in the NC + LPS group, which indicated that diabetes exacerbated LPS‐induced inflammation (Figure 2b–e). We also examined the protein expression of ASC, NLRP3, Caspase‐1, GSDMD, IL‐18, IL‐1β, HMGB1, and SIRT1. More significant downregulation of the histone protein SIRT1 and upregulation of the pyroptosis‐related proteins (ASC, NLRP3, Caspase‐1, GSDMD, IL‐18, IL‐1β, GSDMD‐N, and Cleaved‐Caspase‐1) in the T2DM + LPS group were observed compared to the NC + LPS group and T2DM group, respectively (Figure 2f–n and Supporting Information 1: S1a–e). These results indicate that targeting cardiac pyroptosis holds promise as a therapeutic approach for combating injuries associated with T2DM and sepsis.

### 3.3. LPS‐Induced Pyroptosis in H9c2 Cells Under HG Condition

**Figure 3:**
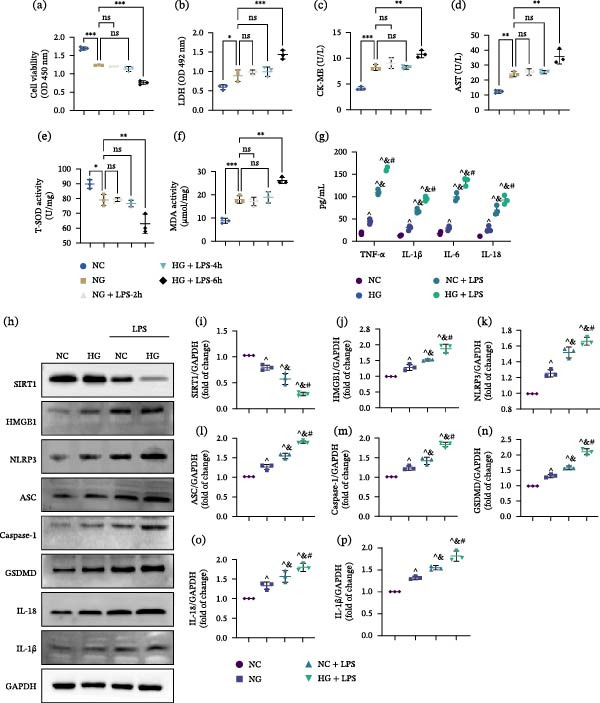
LPS‐induced pyroptosis in vitro under normal and high glucose conditions. (a) Cell viability. (b) Leakage of lactate dehydrogenase (LDH). (c) Creatine kinase‐MB (CK‐MB). (d) Aspartate aminotransferase (AST). (e) Total‐superoxide dismutase (T‐SOD). (f) Malondialdehyde (MDA). (g) Interleukin (IL)‐6, tumor necrosis factor‐α (TNF‐α), IL‐18, and IL‐1β. (h) Representative images of western blot. (i) SIRT1 protein expression. (j) HMGB1 protein expression. (k) NLRP3 protein expression. (l) ASC protein expression. (m) Caspase‐1 protein expression. (n) GSDMD protein expression. (o) IL‐18 protein expression. (p) IL‐1β protein expression. Data are expressed as mean ± SD,  ^∗^ *p*  < 0.05,  ^∗∗^ *p*  < 0.002,  ^∗∗∗^ *p*  < 0.001, *^p* < 0.05 vs. NC group, ^*&*^ *p*  < 0.05 vs. HG group, ^#^ *p*  < 0.01 vs. NC + LPS group, ns: not significant, and *n* = 3 independent biological replicates per group. The grouped gel/blot bands were cropped from the same gel and are clearly delineated in the Supporting Information file.

In vitro, rat heart‐derived H9c2 cells were exposed to HG for 48 h and then subjected to LPS (10μg/ml) for 2, 4, and 6 h in the medium, respectively. As shown in Figure 3, significant reduction of cell viability (Figure 3a) and T‐SOD (Figure 3e), augmentation of LDH leakage (Figure 3b), CK‐MB (Figure 3c), AST (Figure 3d), and MDA (Figure 3f) were observed in the HG + LPS‐6 h group. All these results suggested that significant cellular damage occurred when LPS was administered for 6 h. Therefore, the ensuing experiments were conducted based on this condition.

To further investigate the role of SIRT1 and NLRP3 inflammasome‐dependent pyroptosis in septic myocardial injury under hyperglycemia, TNF‐α, IL‐1β, IL‐6, and IL‐18 were detected using ELISA assays. As depicted in Figure 3g, we found that the HG + LPS group most significantly enhanced H9c2 cell inflammation response, manifested as significant increases in TNF‐α, IL‐1β, IL‐6, and IL‐18. Then, we detected the protein expression of SIRT1 and pyroptosis‐related proteins. As shown in Figure 3h–p and Supporting Information 1: S2a–e, more significant decreased expression in SIRT1 and increased expression of pyroptosis‐related proteins, including HMGB1, ASC, NLRP3, Caspase‐1, GSDMD, IL‐18, IL‐1β, GSDMD‐N, and Cleaved‐Caspase‐1, were seen in the HG + LPS group than the NC + LPS group, which were similar to the changes seen in sepsis‐diabetic mice.

### 3.4. PPF, SAA, or Their Combination Demonstrated Cardiac Protection Against LPS‐Induced Cell Injury Under Hyperglycemic Conditions

**Figure 4:**
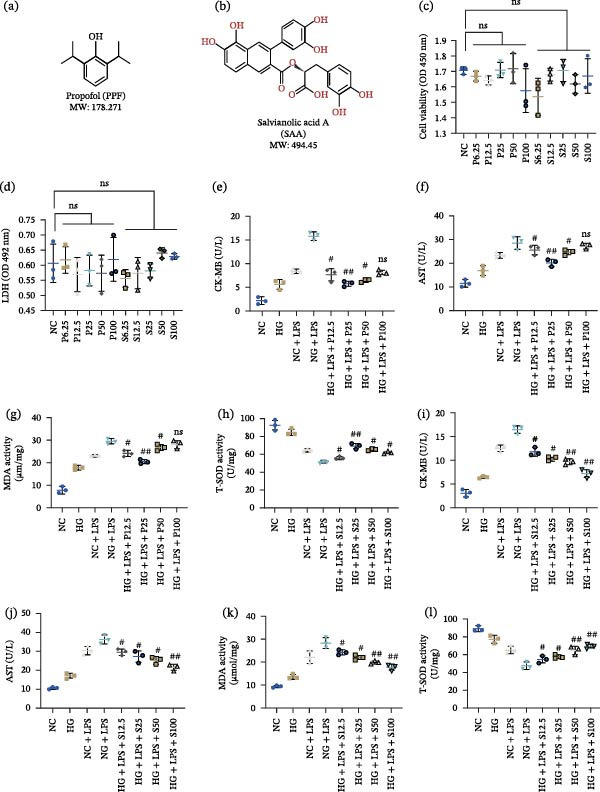
Effects of PPF or SAA against LPS‐induced H9c2 cell injury under hyperglycemia. (a) Chemical structure and molecular weight of PPF. (b) Chemical structure and molecular weight of SAA. (c) Cell viability. (d) Leakage of lactate dehydrogenase (LDH). (e,i) Creatine kinase‐MB (CK‐MB). (f,j) Aspartate aminotransferase (AST). (g,k) Malondialdehyde (MDA). (h,l) Total‐superoxide dismutase (T‐SOD). Data are expressed as mean ± SD. ^#^ *p*  < 0.05 vs. HG + LPS group, ^##^ *p*  < 0.01 vs. HG + LPS group, ns: not significant vs. HG + LPS group, and *n* = 3 independent biological replicates per group.

**Figure 5:**
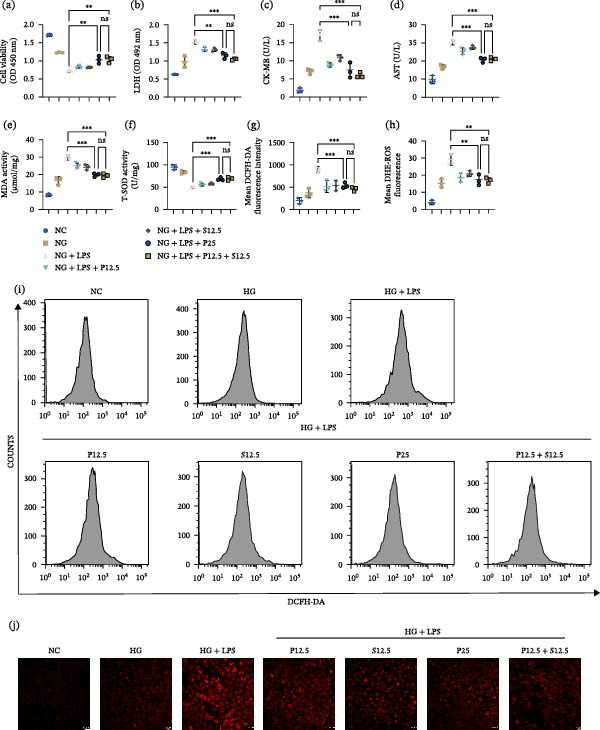
Effects of PPF combined with or without SAA against LPS‐induced cell injury under hyperglycemia. (a) Cell viability. (b) Leakage of lactate dehydrogenase (LDH). (c) Creatine kinase‐MB (CK‐MB). (d) Aspartate aminotransferase (AST). (e) Malondialdehyde (MDA). (f) Total‐superoxide dismutase (T‐SOD). (g) Statistical image of mean 2,7‐dichlorodihydrofluorescein diacetate (DCFH‐DA) fluorescence intensity. (h) Statistical graph of dihydroethidium (DHE) staining. (i) Representative image of DCFH‐DA fluorescence flow cytometry. (j) Representative image of DHE staining (100×, scale bar: 100 μm). Data are shown as mean ± SD;  ^∗^ *p*  < 0.05,  ^∗∗^ *p*  < 0.002,  ^∗∗∗^ *p*  < 0.001, ns: not significant, and *n* = 3 independent biological replicates per group.

It has been reported that PPF attenuates LPS‐induced cardiomyocyte injury by targeting SIRT1‐mediated autophagy [38], and SAA showed a protective effect in various LPS‐induced organ injuries, including heart damage [39]. Both PPF and SAA demonstrate sedative properties and share similar polyphenolic structures (Figure 4a,b), indicating the possibility of synergistic effects when used in combination. Graded from low to high concentration, varying from 6.25, 12.5, 25, 50 to 100 μM, cell viability and LDH leakage assessment (Figure 4c,d) showed that no toxicity was observed in either PPF or SAA for up to 24 h. Besides, PPF and SAA exhibited dose‐dependent protective effects along with upregulation of T‐SOD and downregulation of CK‐MB, AST, and MDA (Figure 4e‐l). PPF demonstrated its most prominent effects at a concentration of 25 μM, and the protective effects of SAA continuously enhanced with increasing concentrations. Based on the aforementioned findings, we hypothesized that a low dose of PPF (12.5 μM, P12.5) in combination with SAA (12.5 μM, S12.5) may provide optimal cardioprotective effects while avoiding the side effects associated with high‐dose PPF (25 μM, P25). As depicted in Figure 5, levels of cell viability (Figure 5a) and T‐SOD (Figure 5f) were increased in the HG + LPS + P12.5 + S12.5 group, while LDH leakage (Figure 5b), CK‐MB (Figure 5c), AST (Figure 5d), and MDA (Figure 5e) were reduced, compared to the HG + LPS group. ROS products, as analyzed by DCDH‐DA fluorescence flow cytometry and DHE staining (Figure 5g–j), were also reduced in the HG + LPS + P12.5 + S12.5 group. It is worth noting that the aforementioned changes between the HG + LPS + P12.5 + S12.5 and HG + LPS + P25 groups were not significantly different.

### 3.5. Coadministration of PPF/SAA Upregulated SIRT1 Expression and Inhibited LPS‐Induced Pyroptosis in H9c2 Cells Under Hyperglycemic Conditions

**Figure 6:**
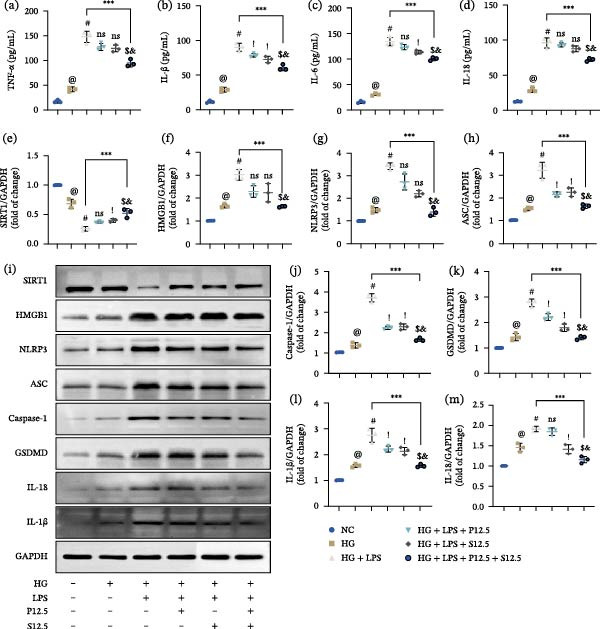
Effects of PPF, with or without SAA, on LPS‐induced pyroptosis in H9c2 cells under hyperglycemic conditions. (a) Tumor necrosis factor (TNF)‐α. (b) Interleukin (IL)‐1β. (c) IL‐6. (d) IL‐18. (e) SIRT1 protein expression. (f) HMGB1 protein expression. (g) NLRP3 protein expression. (h) ASC protein expression. (i) Representative images of western blot. (j) Caspase‐1 protein expression. (k) GSDMD protein expression. (l) IL‐1β protein expression. (m) IL‐18 protein expression. Data are expressed as the mean ± SD, *n* = 3 independent biological replicates per group.  ^∗∗∗^ *p*  < 0.001, ^@^ *p*  < 0.05 vs. NC group, ^#^ *p*  < 0.05 vs. HG group, ^!^ *p*  < 0.05 vs. HG + LPS group, ^$^ *p*  < 0.05 vs. HG + LPS + P12.5 group, ^&^ *p*  < 0.05 vs. HG + LPS + S12.5 group, and ns: not significant vs. HG + LPS group. The grouped gel/blot bands were cropped from the same gel and are clearly delineated in the Supporting Information file.

Previous studies have demonstrated that SAA or PPF exerts protective effects against pyroptotic cell death in endothelial and macrophage cells [26, 27]; however, the effects of these compounds on cell injury induced by HG and LPS remain unclear. In this study, low doses of PPF, SAA, or their combination were, respectively, administered to H9c2 cells in conjunction with LPS. Pyroptotic‐related inflammatory cytokines, including IL‐18, IL‐6, TNF‐α, and IL‐1β, in the treatment group (HG + LPS + P12.5 + S12.5 group) were lower than in the HG + LPS group (Figure 6a–d). Meanwhile, as illustrated in Figure 6e–m and S3a‐e, the protein expression of SIRT1 was markedly elevated, whereas the protein expression levels of ASC, NLRP3, Caspase‐1, GSDMD, HMGB1, IL‐18, IL‐1β, GSDMD‐N, and Cleaved‐Caspase‐1 were significantly reduced in the HG + LPS + P12.5 + S12.5 group as compared to the HG + LPS group. It was noted that, in the in vitro studies, the levels of the abovementioned parameters did not significantly differ between the PPF (HG + LPS + P12.5 group) and the SAA (HG + LPS + S12.5 group) treatment alone groups, nor did they differ significantly from those in the group with PPF and SAA coadministration (HG + LPS + P12.5 + S12.5 group).

### 3.6. Coadministration of PPF and SAA Mitigated Cardiac Dysfunction and Pyroptosis Following LPS‐Induced Damage In Vivo

**Figure 7:**
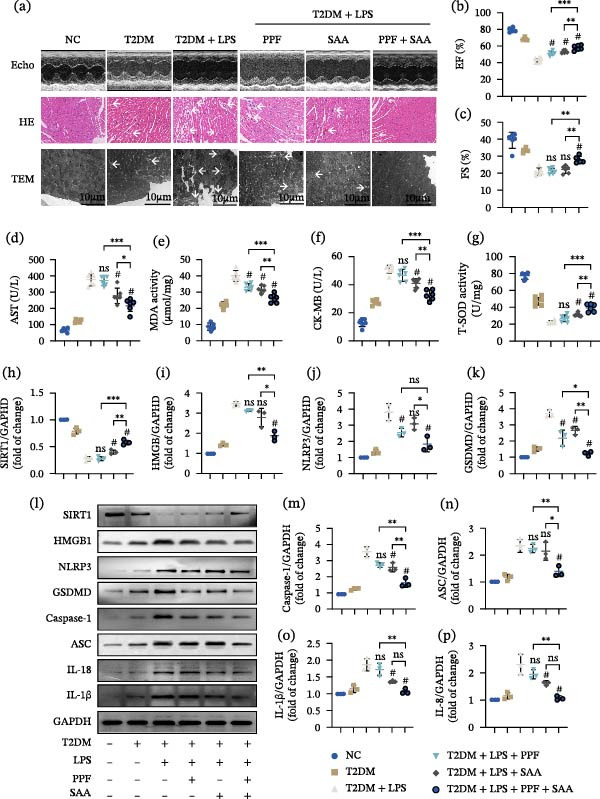
Effects of PPF combined with or without SAA on LPS‐induced cardiac dysfunction in diabetic mice. (a) Echo: the representative images of the echocardiogram; HE: representative histological images from HE staining heart sections (200×, scale bar: 100 μm); TEM: the results of transmission electron microscopy (scale bar: 100 μm), arrows indicate characteristic changes. (b) Ejection fraction (EF%). (c) Fractional shortening (FS%). (d) Aspartate aminotransferase (AST). (e) Malondialdehyde (MDA). (f) Creatine kinase‐MB (CK‐MB). (g) Total‐superoxide dismutase (T‐SOD). (h) SIRT1 protein expression. (i) HMGB1 protein expression. (j) NLRP3 protein expression. (k) GSDMD protein expression. (l) Representative images of western blots. (m) Caspase‐1 protein expression. (n) ASC protein expression. (o) IL‐1β protein expression. (p) IL‐18 protein expression. Data are expressed as the mean ± SD, *n* = 6 per group (a–g) and *n* = 3 per group (h–p). ^#^ *p*  < 0.05 vs. T2DM + LPS group,  ^∗^ *p*  < 0.05,  ^∗∗^ *p*  < 0.002,  ^∗∗∗^ *p*  < 0.001, and ns: not significant vs. T2DM + LPS group. The grouped gel/blot bands were cropped from the same gel and are clearly delineated in the Supporting Information file.

Since the coadministration of low doses of PPF and SAA yielded beneficial outcomes in vitro under HG conditions, we replicated this approach in vivo in diabetic mice. The dosages of both PPF and SAA were consistent with those used in our previous research [28], ensuring comparability in the current experiments. As shown in Figure 7a–c, heart function has been improved in the drug intervention groups as compared to that in the T2DM + LPS group, as evidenced by increased EF% and FS% values compared to diabetic‐septic mice, which was further significantly improved in the joint PPT and SAA treatment (T2DM + LPS + PPF + SAA) group. The T2DM + LPS + PPF + SAA group exhibited a significant reduction in inflammatory cell infiltration and cell swelling and a more orderly arrangement of cells (Figure 7a; HE). Additionally, after drug intervention, there was a reduction in cardiac injury markers (Figure 7d,f) and lipid peroxidation products (Figure 7e), along with an increase in the antioxidant enzyme T‐SOD (Figure 7g), further illustrating the benefits of using PPF and SAA either in combination or individually. Of note, more prominent beneficial effects were seen in the T2DM + LPS + PPF + SAA group as compared to those in the T2DM + LPS + PPF group.

Regarding pyroptosis, TEM was performed to conduct further observations on the myocardial tissue. The formation of dense materials within cells was reduced, mitochondrial damage induced by LPS was mitigated, and also reduced were the subtle vesicles formed around the cell membrane in the PPF and/or SAA treatment groups as compared with the T2DM + LPS group (Figure 7a; TEM). Additionally, the protein expression of SIRT1 (Figure 7h) was significantly upregulated, whereas the protein expression levels of ASC, NLRP3, Caspase‐1, GSDMD, HMGB1, IL‐18, IL‐1β, GSDMD‐N, and Cleaved‐Caspase‐1 were markedly decreased in the T2DM + LPS + PPF + SAA group as compared to that in the T2DM + LPS group (Figure 7h–p and Supporting Information 1: S4a–e).

### 3.7. Inhibition of SIRT1 Abrogated the Cardioprotective Effects of the Coadministration of PPF/SAA Both In Vivo and In Vitro

**Figure 8:**
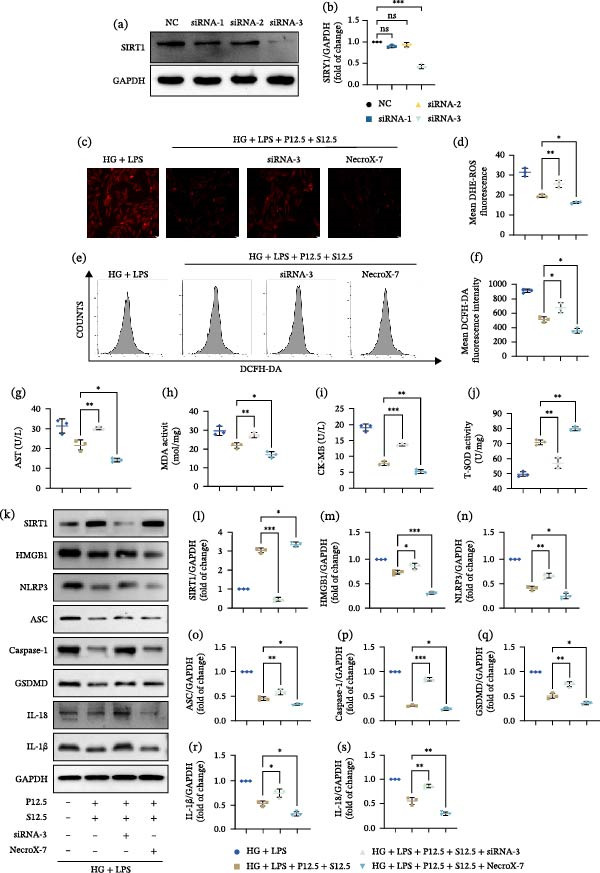
Silencing of SIRT1 abrogated the protective effects of PPF combined with SAA against LPS‐ and HG‐induced cellular injury and pyroptosis in vitro. (a) Representative image of SIRT1 silence. (b) SIRT1 protein expression. (c) Representative image of dihydroethidium (DHE) staining (100×, scale bar: 100 μm). (d) Statistical graph of DHE staining. (e) Representative image of 2, 7‐dichlorodihydrofluorescein diacetate (DCFH‐DA) fluorescence flow cytometry. (f) Statistical image of mean DCFH‐DA fluorescence intensity. (g) Aspartate aminotransferase (AST). (h) Malondialdehyde (MDA). (i) Creatine kinase‐MB (CK‐MB). (j) Total‐superoxide dismutase (T‐SOD). (k) Representative images of western blots. (l) SIRT1 protein expression. (m) HMGB1 protein expression. (n) NLRP3 protein expression. (o) ASC protein expression. (p) Caspase‐1 protein expression. (q) GSDMD protein expression. (*r*) IL‐1β protein expression. (s) IL‐18 protein expression. Data are shown as mean ± SD, *n* = 3 independent biological replicates per group.  ^∗^ *p*  < 0.05,  ^∗∗^ *p*  < 0.002,  ^∗∗∗^ *p*  < 0.001, and ns: not significant. The grouped gel/blot bands were cropped from the same gel and are clearly delineated in the Supporting Information file.

**Figure 9:**
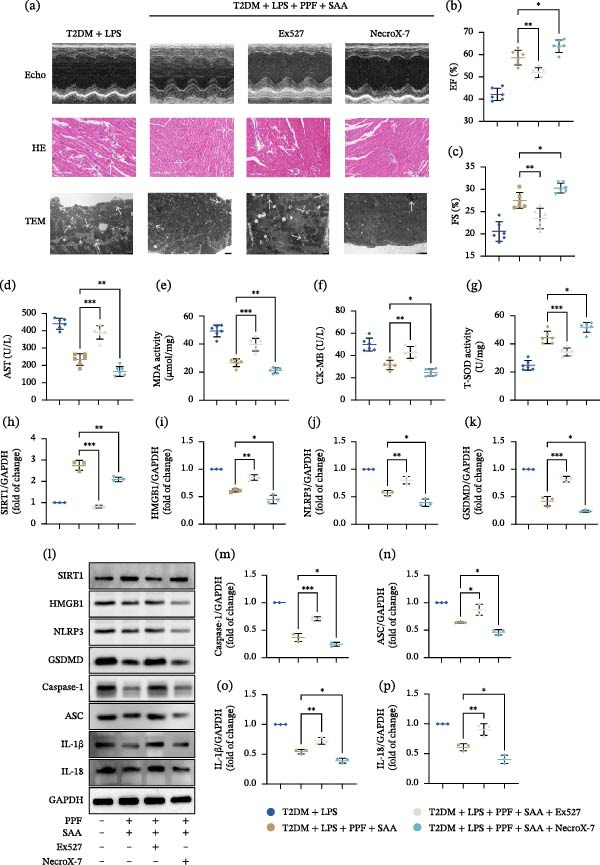
Changes in pyroptosis following the inhibition of SIRT1 or HMGB1 in sepsis‐diabetic mice. (a) Echo: the representative images of the echocardiogram; HE: Representative histological images from HE staining heart sections (200×, scale bar: 100 μm); TEM: The results of transmission electron microscopy (scale bar: 100 μm), arrows indicate characteristic changes. (b) Ejection fraction (EF%). (c) Fractional shortening (FS%). (d) Aspartate aminotransferase (AST). (e) Malondialdehyde (MDA). (f) Creatine kinase‐MB (CK‐MB). (g) Total‐superoxide dismutase (T‐SOD). (h) SIRT1 protein expression. (i) HMGB1 protein expression. (j) NLRP3 protein expression. (k) GSDMD protein expression. (l) Representative images of western blots. (m) Caspase‐1 protein expression. (n) ASC protein expression. (o) IL‐1β protein expression. p) IL‐18 protein expression. Data are expressed as the mean ± SD, *n* = 6 per group (a–g) and *n* = 3 per group (h–p).  ^∗^ *p*  < 0.05,  ^∗∗^ *p*  < 0.002,  ^∗∗∗^ *p*  < 0.001, and ns: not significant. The grouped gel/blot bands were cropped from the same gel and are clearly delineated in the Supporting Information file.

To validate the association between LPS‐induced injury and the SIRT1‐HMGB1‐dependent pyroptosis axis and to elucidate the mechanism of action of the combined use of PPF and SAA, we meticulously designed and synthesized three distinct siRNA sequences targeting SIRT1. Additionally, we introduced the HMGB1 inhibitor NecroX‐7 to modulate the HMGB1 expression. When SIRT1 was knocked down in H9c2 cells using three distinct siRNA‐SIRT1 sequences, transfection with siRNA‐3 led to the most significant reduction in SIRT1 protein expression, as demonstrated in Figure 8a,b. Then, we evaluated the efficacy of PPF in combination with SAA on LPS‐induced cell injury under HG conditions in H9c2 cells following siRNA‐3 transfection and NecroX‐7 respectively. As shown in Figure 7c–j, the levels of ROS products and cardiac injury were significantly increased in the HG + LPS + P12.5 + S12.5 + siRNA‐3 group compared to those in the HG + LPS + P12.5 + S12.5 group. Also, the cardioprotective effects of the PPF and SAA combination were significantly enhanced through the inhibition of HMGB1 expression. As illustrated in Figure 8, the expression levels of pyroptosis‐related proteins (Figure 8n–s and Supporting Information 1: S5a–e) were significantly elevated compared to groups without SIRT1 knockdown. Of note, HMGB1 protein expression was increased in the HG + LPS + P12.5 + S12.5 + siRNA‐3 group compared to the HG + LPS + P12.5 + S12.5 group (Figure 8m). These findings collectively indicate that the coadministration of PPF and SAA alleviated the cell damage and pyroptosis induced by LPS and HG. However, the antipyroptosis effects of the combined use of PPF and SAA were abolished when SIRT1 was silenced, while the inhibition of HMGB1 further potentiated these beneficial effects of joint PPF and SAA treatments. In vivo, SIRT1 (Ex527) and HMGB1 inhibitors were administered to sepsis‐diabetic mice. Ex527 treatment neutralized the beneficial effects of joint PPF and SAA treatment on cardiac function, as evidenced by a decline in EF% and FS% (Figure 9a; Echo, b–c) and induced greater inflammatory cell infiltration and subtle vesicles, as shown by HE staining and TEM scanning (Figure 9a; HE and TEM). The levels of serum MDA, AST, and CK‐MB were also elevated, while T‐SOD was reduced (Figure 9d–g). Pyroptosis‐related proteins (Figure 9i–p and Supporting Information 1: a–e) analyzed by western blotting exhibited significant increases following Ex527 intervention,despite the presence of PPF and SAA. On the other hand, inhibition of HMGB1 with Necrox‐7 potentiated the beneficial effects of PPF + SAA treatment against pyroptosis. Based on the aforementioned in vitro and in vivo experimental results, which consistently demonstrated that the coadministration of PPF and SAA exhibited cardioprotective effects by regulating the SIRT1/HMGB1 pathway to inhibit pyroptosis.

## 4. Discussion

The intricate process by which diabetes exacerbates sepsis‐induced cardiac dysfunction is multifaceted, involving elevated levels of complement proteins, mitochondrial dysfunction and redox imbalance, impaired calcium homeostasis, and neutrophil dysfunction, among other factors [40]. In this study, it was observed that LPS had a detrimental effect on myocardial function in HFD‐fed and STZ‐induced diabetic mice that was concomitant with a reduction in cardiac SIRT1 levels and an elevation in pyroptosis‐related proteins. Then, we have illustrated that low concentration of PPF combined with SAA attenuated oxidative stress response and ameliorated LPS‐induced cell injury by targeting the SIRT1/HMGB1 pathway to inhibit NLRP3‐dependent pyroptosis under hyperglycemia in vitro and in vivo.

Diabetes is associated with increased susceptibility to infection and sepsis, but the impact of diabetes on various organ functions from sepsis is still controversial. Prior studies have shown that diabetes does not have a significant effect on mortality rates or disrupt homeostatic and inflammatory responses in patients with severe sepsis across multiple intensive care units in various countries [41]. However, a recent population‐based cohort study has revealed interesting results, suggesting that individuals with preexisting diabetes who have survived sepsis are at a significantly higher long‐term risk of major cardiovascular events compared to sepsis survivors without diabetes [42]. Studies have shown that the impact of hyperglycemia and high glycemic variability (GV) on mortality increases with the severity of sepsis [43]. Additionally, high GV within 72 h in patients with sepsis is independently associated with an increased risk of sepsis‐related disseminated intravascular coagulation. This association is not influenced by preexisting diabetes [44]. Furthermore, a lower admission blood glucose level has been reported to be associated with an increased risk of poor prognosis [45]. The reasons for the different outcomes between these studies are unclear but may relate to differences in the pathophysiological mechanisms, such as inflammatory response, dysregulated immune pathways, etc. [6].

Pyroptosis, an inflammatory mode of cellular death, has been implicated in a multitude of infectious and noninfectious diseases and is triggered by various pathological stimuli, including oxidative stress, hyperglycemia, and inflammation [46, 47]. The NLRP3 inflammasome, consisting of NLRP3, ASC, and Caspase‐1, plays a crucial role in the innate immune response. Once activated, NLRP3 assembles the abovementioned cytosolic immune complex that motivates caspase‐1 and then cleaves GSDMD, which facilitates the processing and secretion of proinflammatory cytokines, specifically IL‐1β and IL‐18, while also inducing pyroptosis [48]. Our findings are in accord with recent studies, wherein it was observed that natural herbs/medicines could decrease ROS release induced by LPS and alleviate cardiovascular dysfunction by inhibiting the direct activation of the NLRP3 inflammasome in vivo and in vitro [49, 50]. Moreover, a growing body of evidence has demonstrated that the mitigation of cardiac pyroptosis yields advantageous outcomes in the context of DCM [50, 51]. Interestingly, a recent study has demonstrated that the knockdown of the NLRP3 gene significantly suppresses DCM‐induced myocardial pyroptosis and ferroptosis [52]. This is particularly relevant considering that DCM is characterized by a proinflammatory mechanism, thus indicating the potential of targeting cardiac pyroptosis as a therapeutic strategy for DCM. As for sepsis, inhibiting excessive pyroptosis in cardiomyocytes appears to be a potential and critical therapeutic strategy for sepsis patients as significant pyroptosis in the cardiac tissue of septic mice has been verified through mRNA sequence analysis [53]. However, the currently available studies are constrained by notable limitations, primarily stemming from the absence of precise elucidation of the mechanisms underlying cardiac pyroptosis in both T2DM and sepsis.

As a member of class III histone/protein deacetylases, SIRT1 regulates key metabolic processes, including stress resistance, inflammation, pyroptosis, and apoptosis, through the deacetylation of different substrates [54]. SIRT1 has been reported to inhibit the activity of HMGB1. Both are related to the occurrence and development of inflammation and associated diseases but show an antagonistic relationship in directly controlling inflammation. Wei et al. [17] demonstrated that syringaresinol (a natural abstract that possesses anti‐inflammatory properties) improved cardiac function and alleviated myocardial injury in mice that were subjected to cecal ligation and puncture via the SIRT1/NLRP3/GSDMD pathway Ding et al’s [55] findings indicated that Panaxynol showed cardioprotective effects in IRI‐induced apoptosis and pyroptosis through regulating the HMGB1/TLR4/NF‐kB pathway and inhibiting NLRP3 inflammasome stimulation. An in vitro study demonstrated that the depletion of SIRT1 effectively nullified the advantageous impact of growth differentiation factor 11 in mitigating H9c2 cell injury induced by HG and palmitate, achieved by attenuating oxidative injury and apoptosis [56]. A recent investigation demonstrates that the silencing of SIRT1 could alleviate cardiac ischemia‐reperfusion injury‐associated pyroptosis via the HMGB1/NF‐κB/NLRP3 axis, indicating that SIRT1 functions as a double‐edged sword in this context [57]. We, thus, hypothesized that there is also an interaction between pyroptosis and the SIRT1/HMGB1 pathway in diabetic‐sepsis mice and subsequently observed alterations in the expression of SIRT1, HMGB1, and pyroptosis‐related proteins *in vivo* and further substantiated their relationship in cellular and animal models.

Septic patients and diabetic patients are less tolerable to cardiovascular procedures [41]. PPF has been widely utilized in various endoscopic examinations and surgical procedures and studied due to its protective properties, such as the elimination of ROS, inhibition of cell death, maintenance of mitochondrial function, and reduction of inflammatory mediators [24, 58]. However, overdose of PPF may initiate cell death of pyroptosis through the NLRP3/caspase‐1 signal [26], and high doses of PPF have been shown to cause detrimental and potentially fatal clinical consequences such as respiratory depression and cardiac arrest [58]. It is intriguing to know that the main active component of Salviae miltiorrhizae radix et rhizome (a valuable Chinese medicine), SAA, has protective effects against cardiovascular disease and diabetes. Zhu et al. [27] revealed that SAA could ameliorate atherosclerosis in Western diet‐fed STZ‐induced diabetic *ApoE* ^−/−^ mice by regulating endothelial pyroptosis via the PKM2/PKR/NLRP3 inflammasome signaling pathway, and the combined utilization of in silico analysis and experimental validation revealed that SAA exerts its anti‐inflammatory properties through the p38‐HO‐1 pathway in LPS‐stimulated RAW264.7 cells [59]. SAA possesses a wider and safer medication direction; meanwhile, PPF and SAA show partial structural similarity in terms of their phenol structure [25, 60]. This article addresses the controversy and inconsistency surrounding the cytotoxic effects of PPF, especially when it is used at higher concentrations, and its impracticality for its usage in critically ill patients with multiple comorbidities. Given the clinical heterogeneity of sepsis, further studies should assess the robustness of this combination therapy across different sepsis phenotypes—such as distinguishing between sepsis‐induced myocardial dysfunction in preexisting diabetes vs. stress‐induced hyperglycemia—and explore the equilibrium of oxidative stress under varying pathophysiological conditions.

A limitation of this study is the use of male mice only. Future studies should include female subjects to determine whether the synergistic protective effects of PPF and SAA on LPS‐induced myocardial pyroptosis are influenced by sex‐related hormonal differences. Additionally, our current research is restricted to a single‐cell line and a mouse model of sepsis following diabetes. The translation of the combination of PPF and salvianolic acid into clinical practice remains a significant challenge. Finally, while our focus was on cardiac protection, the systemic nature of sepsis necessitates evaluating the efficacy of this combination in other vital organs, particularly the lung and kidney, to fully assess its therapeutic potential. Bridging the gap from preclinical models to clinical applications remains a significant challenge that will require rigorous multicenter validation.

## 5. Conclusion

The findings of our current study provide evidence supporting the supplementary use of SAA to enhance the efficacy of low‐dose PPF in cardiac protection. This approach shows promise as a potential therapeutic intervention for individuals with T2DM and sepsis by modulating the SIRT1‐HMGB1‐dependent pyroptosis pathway.

**Nomenclature**
T2DM: Type 2 diabetes mellitus
DCM: Diabetes cardiomyopathy
ROS: Reactive oxygen species
IL: Interleukin
NLRP3: Nod‐like receptor protein‐3
LPS: Lipopolysaccharide
SIRT1: Silent information regulator 1
NAD: Nicotinamide adenosine dinucleotide
MIRI: Myocardial ischemia‐reperfusion injury
H/R: Hypoxia–reoxygenation
HMGB1: High‐mobility group box 1
PPF: Propofol
SAA: Salvianolic acid A
HFD: High‐fat diet
STZ: Streptozotocin
IP: Intraperitoneal injection
EF%: Ejection fraction
FS%: Fractional shortening
H&E: Hematoxylin–eosin
TEM: Transmission electron microscopy
HG: High glucose
DHE: Dihydroethidium
DCFH‐DA: 2,7‐Dichlorodihydrofluorescein diacetate
AST: Aspartate transaminase
CK‐MB: Creatine phosphokinase‐MB
TNF‐α: Tumor necrosis factor‐α
T‐SOD: Total‐superoxide dismutase
MDA: Malondialdehyde
GV: Glycemic variability.

## Author Contributions

This study was conceptualized and designed by Anyuan Zhang, Ronghui Han, and Zhengyuan Xia. Methodology was developed by Anyuan Zhang, Ronghui Han, and Kaijia Han. Investigation and formal analysis were performed by Anyuan Zhang, Ronghui Han, Kaijia Han, Ting Li, Jiaqi Zhou, Jiajia Chen, Yongyan Wang, Weiyi Xia, and Jianyu Zhu. Anyuan Zhang and Ronghui Han were responsible for visualization and validation and drafting the original manuscript. Wangning Shangguan, Stanley Sau Ching Wong, Guanghua Chen, and Zhengyuan Xia supervised the study and critically reviewed and edited the manuscript. Zhengyuan Xia also provided resources and secured funding.

## Funding

This study was supported by a grant of the National Natural Science Foundation of China (NSFC) (Grant 82270306).

## Disclosure

All authors have read and approved the final manuscript.

## Ethics Statement

This study was approved by the Experimental Animal Ethics Committee of the Affiliated Hospital of Guangdong Medical University (Approval Number AHGDMU‐LAC‐B‐202205‐0078). The animal experiments were conducted under the License Number SYXK (Yue)‐2022‐0286. All procedures were performed in accordance with the guidelines and regulations approved by the committee. The authors complied with the ARRIVE guidelines, and all experiments adhered to the ARRIVE guidelines. The ARRIVE checklist is provided in the Supporting Information.

## Consent

The authors have nothing to report.

## Conflicts of Interest

The authors declare no conflicts of interest.

## Supporting Information

Additional supporting information can be found online in the Supporting Information section.

## Supporting information


**Supporting Information 1** Figures: Full‐length Western blot gels for GSDMD and Caspase‐1, including intact proteins and their *N*‐terminal/cleaved fragments.

**Supporting Information 2** Complete Western blot gels showing all original replicates (*n* = 3) for the experiments described in this study.

## Data Availability

Data collected and analyzed for the study are available from the corresponding author upon reasonable request.
