# Supplementary material for: Propofol and Salvianolic Acid a Synergistically Attenuate LPS‐Induced Myocardial Pyroptosis in Diabetic Mice via the SIRT1/HMGB1 Pathway
Source: Mediators Inflamm. 2026 Jul 7;2026:6298056. doi: 10.1155/mi/6298056 (PMC13338769; doi:10.1155/mi/6298056)
Supplement: Supplementary file 1 — Supporting Information 1 Figures: Full‐length Western blot gels for GSDMD and Caspase‐1, including intact proteins and their N‐terminal/cleaved fragments. [file MI-2026-6298056-s001.pdf]

S1: supplement western blot for figure 2

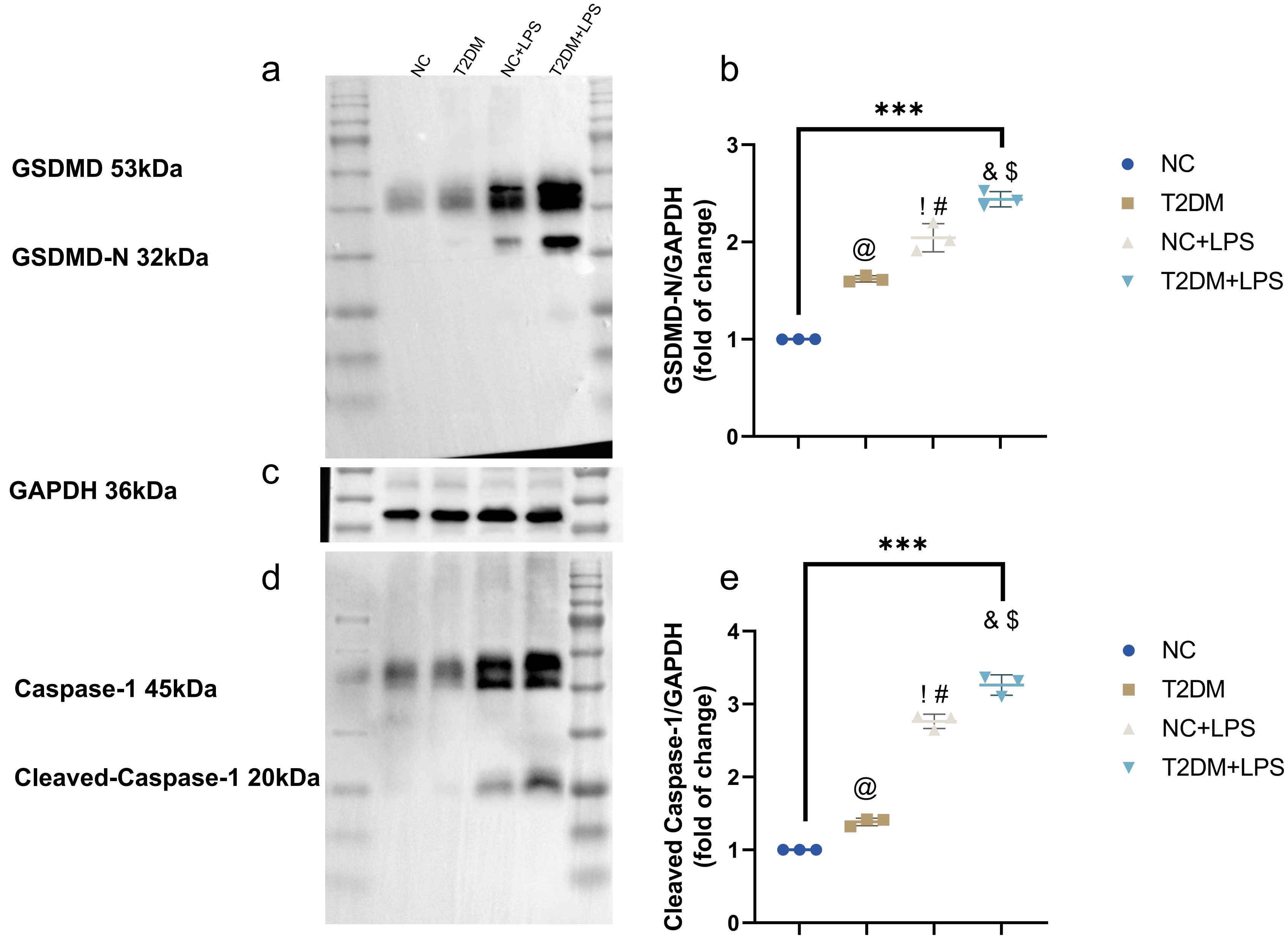

S1: Entire gels including full-length proteins as well as N-terminal or cleaved fragments in GSDMD and Caspase-1.

a) Representative image of western blot (GSDMD and GSDMD-N). b) GSDMD-N protein expression. c) Representative image of GAPDH. d) Representative image of western blot (Caspase-1 and Cleaved-Caspase-1). e) Cleaved-Caspase-1 protein expression. Data are expressed as the mean±SD, n=3 per group. $^{@}p < 0.05$ vs. NC group, $^{!}p < 0.05$ vs. T2DM group, $^{\#}p < 0.05$ vs. NC group, $^{\$}p < 0.05$ vs. T2DM group, $^{\&}p < 0.05$ vs. NC+LPS group, $^{***}p < 0.001$.

S2: supplement western blot for figure 3

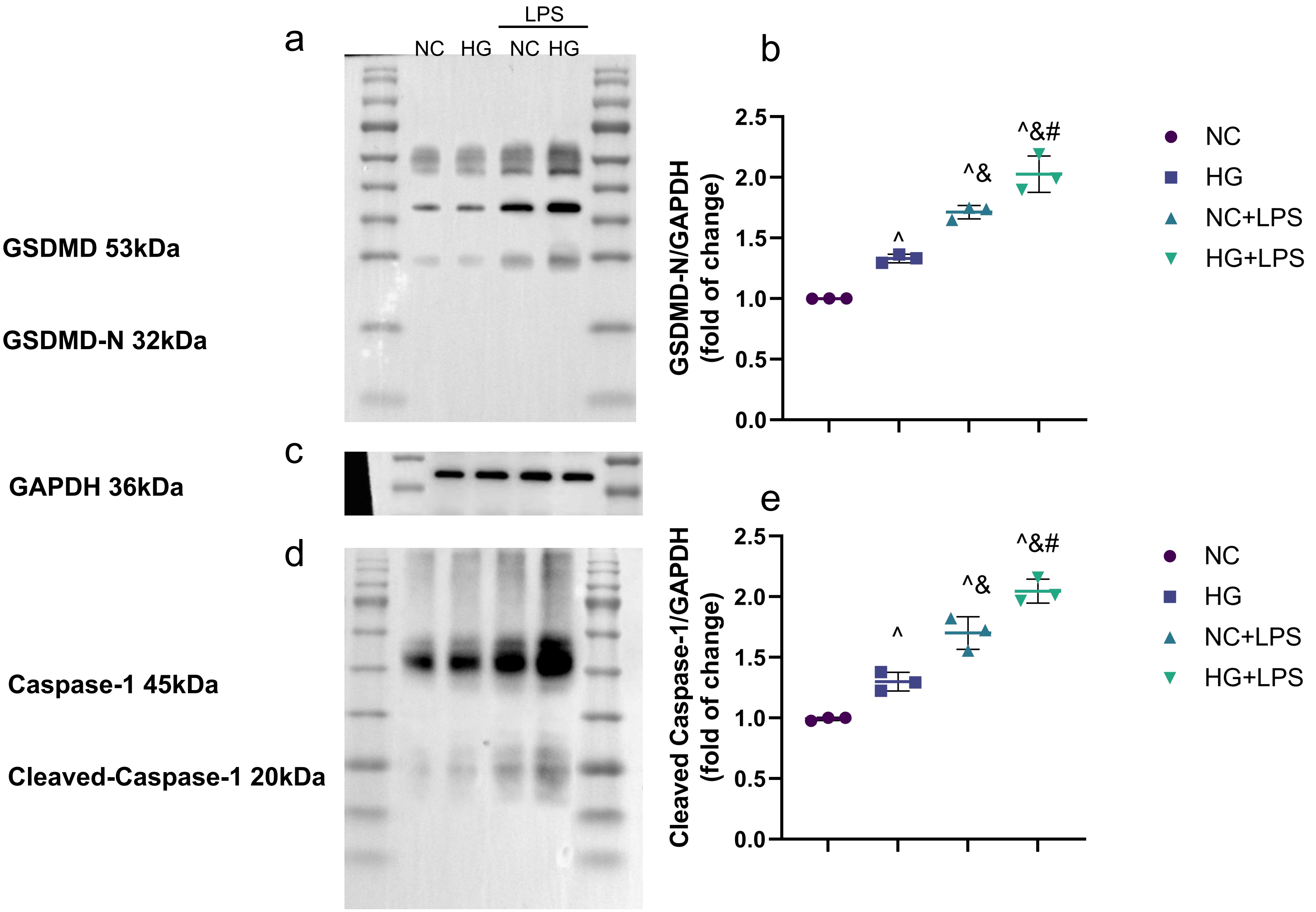

S2: Entire gels including full-length proteins as well as N-terminal or cleaved fragments in GSDMD and Caspase-1.

a) Representative image of western blot (GSDMD and GSDMD-N). b) GSDMD-N protein expression. c) Representative image of GAPDH. d) Representative image of western blot (Caspase-1 and Cleaved-Caspase-1). e) Cleaved-Caspase-1 protein expression. Data are expressed as the mean±SD, n=3 per group.^p< 0.05 *vs* NC group, $^{\&}p$<0.05 *vs* HG group, $^{\#}p$<0.01 *vs* NC+LPS group, ns: not significant, n = 3 per group.

S3: supplement western blot for figure 6

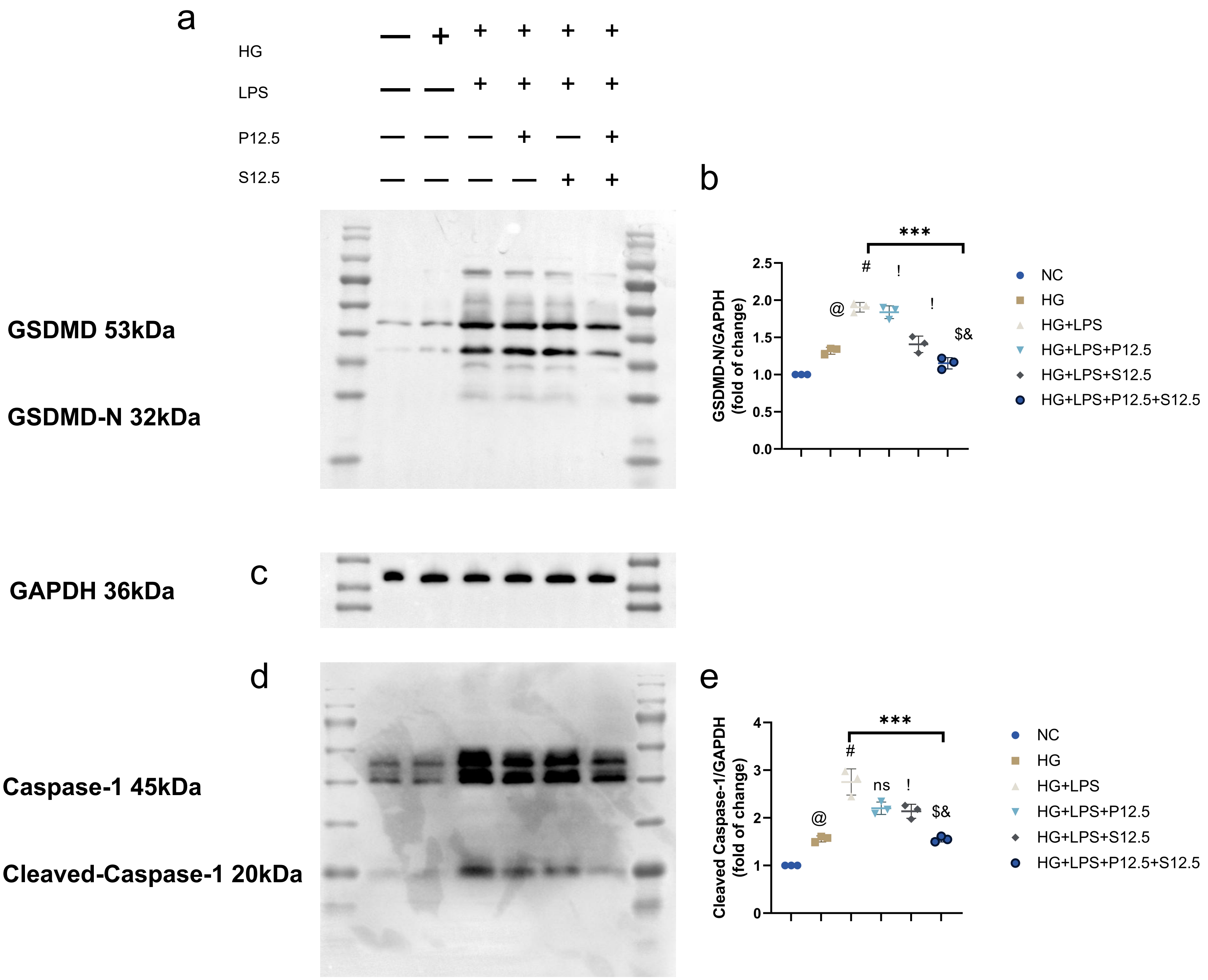

S3：Entire gels including full-length proteins as well as N-terminal or cleaved fragments in GSDMD and Caspase-1.

a) Representative image of western blot (GSDMD and GSDMD-N). b) GSDMD-N protein expression. c) Representative image of GAPDH. d) Representative image of western blot (Caspase-1 and Cleaved-Caspase-1). e) Cleaved-Caspase-1 protein expression. Data are expressed as the mean±SD, n=3 per group, $***p < 0.001$, $^{@}p < 0.05$ *vs.* NC group, $^{\#}p < 0.05$ *vs.* HG group, $^{!}p < 0.05$ *vs.* HG+LPS group, $^{\$}p < 0.05$ *vs.* HG+LPS+P12.5 group, $^{\&}p < 0.05$ *vs.* HG+LPS+S12.5 group, ns:not significant, *vs.* HG+LPS group.

S4: supplement western blot for figure 7

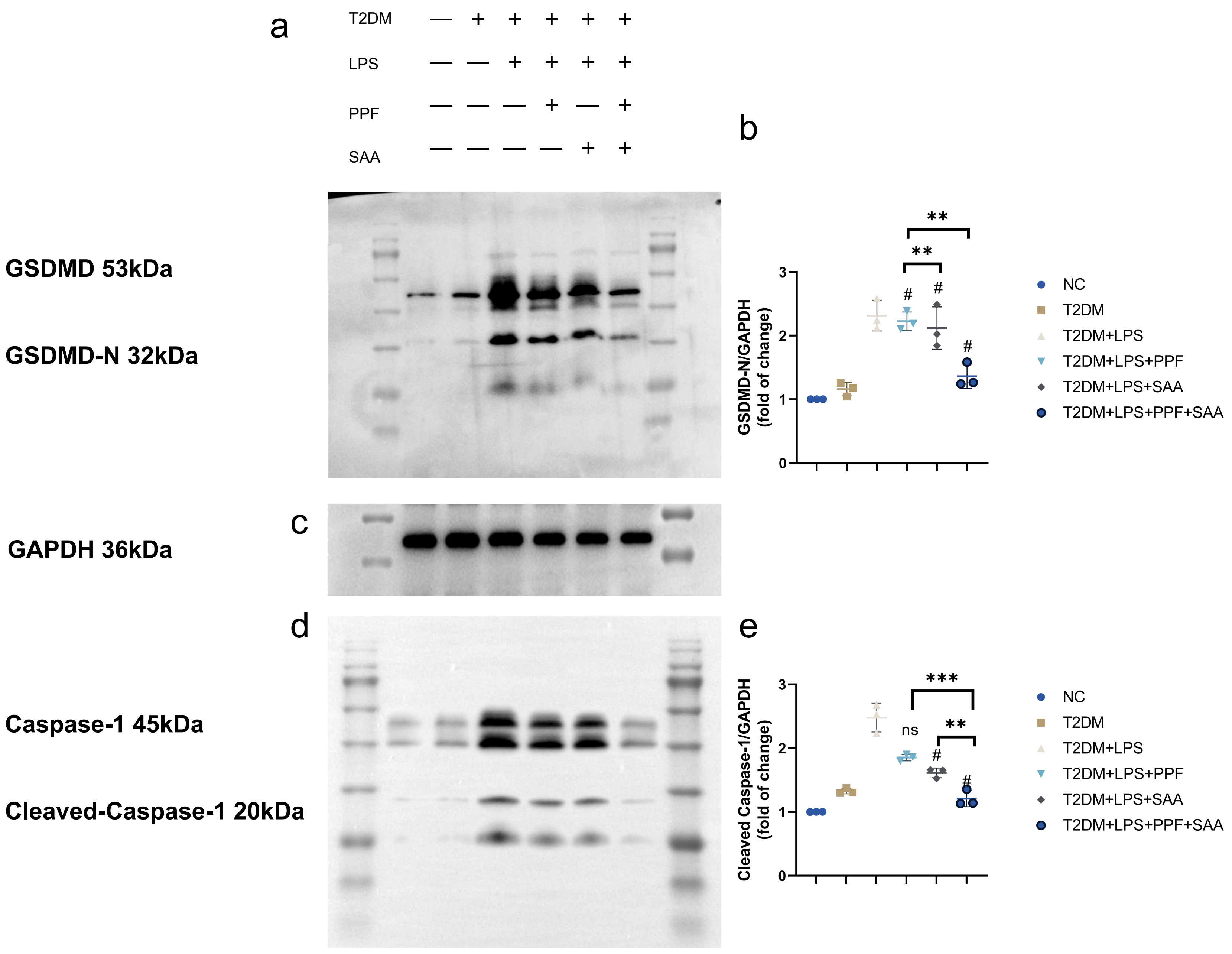

S4: Entire gels including full-length proteins as well as N-terminal or cleaved fragments in GSDMD and Caspase-1.

a) Representative image of western blot (GSDMD and GSDMD-N). b) GSDMD-N protein expression. c) Representative image of GAPDH. d) Representative image of western blot (Caspase-1 and Cleaved-Caspase-1). e) Cleaved-Caspase-1 protein expression. Data are expressed as the mean±SD, n=3 per group. $^{\#}p< 0.05$ *vs.* T2DM+LPS group, $^{*}p< 0.05$, $^{**}p< 0.002$ $^{***}p< 0.001$, ns: not significant, *vs.* T2DM+LPS group.

S5: supplement western blot for figure 8

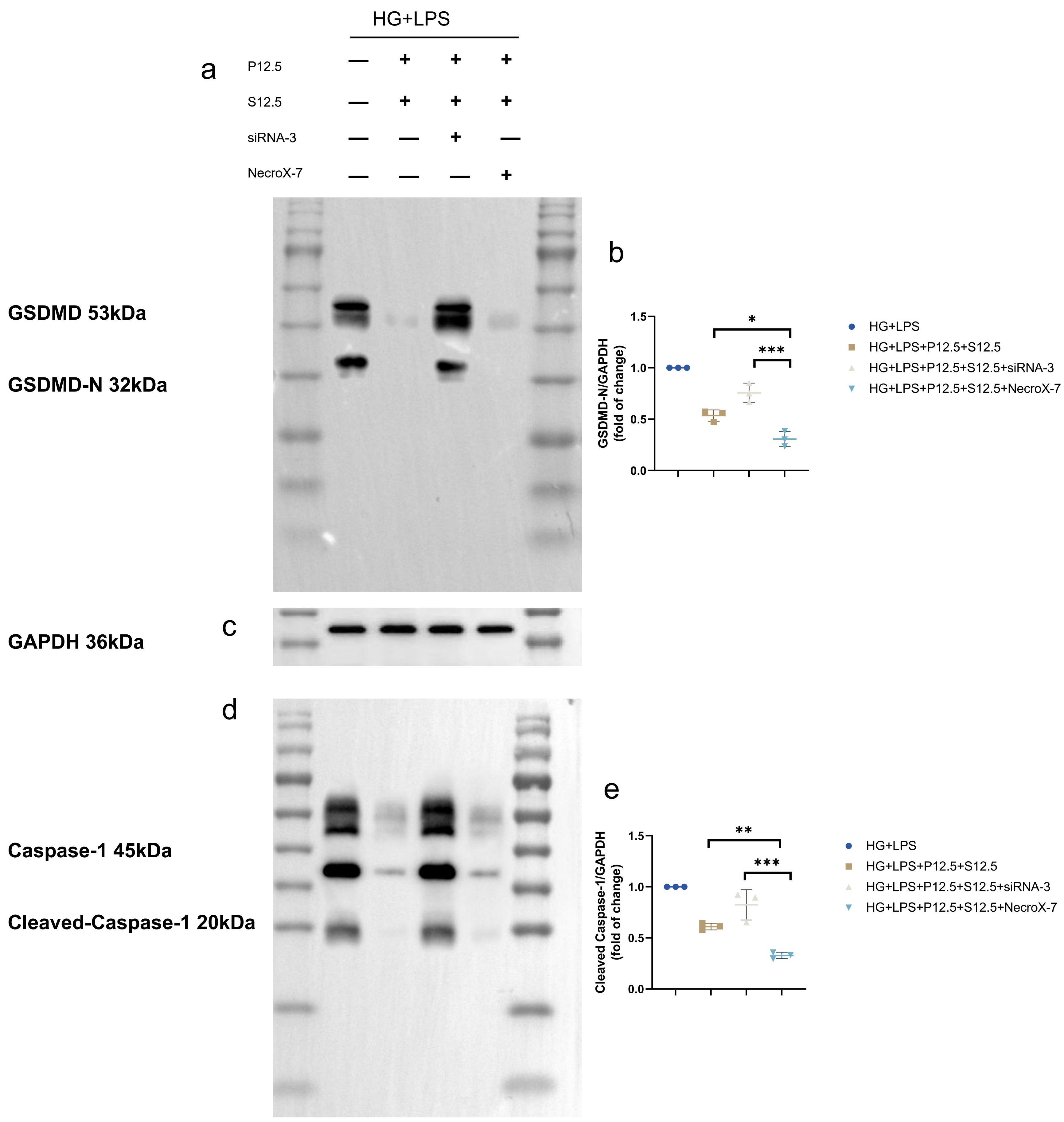

S5： Entire gels including full-length proteins as well as N-terminal or cleaved fragments in GSDMD and Caspase-1.

a) Representative image of western blot (GSDMD and GSDMD-N). b) GSDMD-N protein expression. c) Representative image of GAPDH. d) Representative image of western blot (Caspase-1 and Cleaved-Caspase-1). e) Cleaved-Caspase-1 protein expression. Data are expressed as the mean±SD, n=3 per group. $^{*}p < 0.05$, $^{**}p < 0.002$, $^{***}p < 0.001$, ns: not significant.

S6: supplement western blot for figure 9

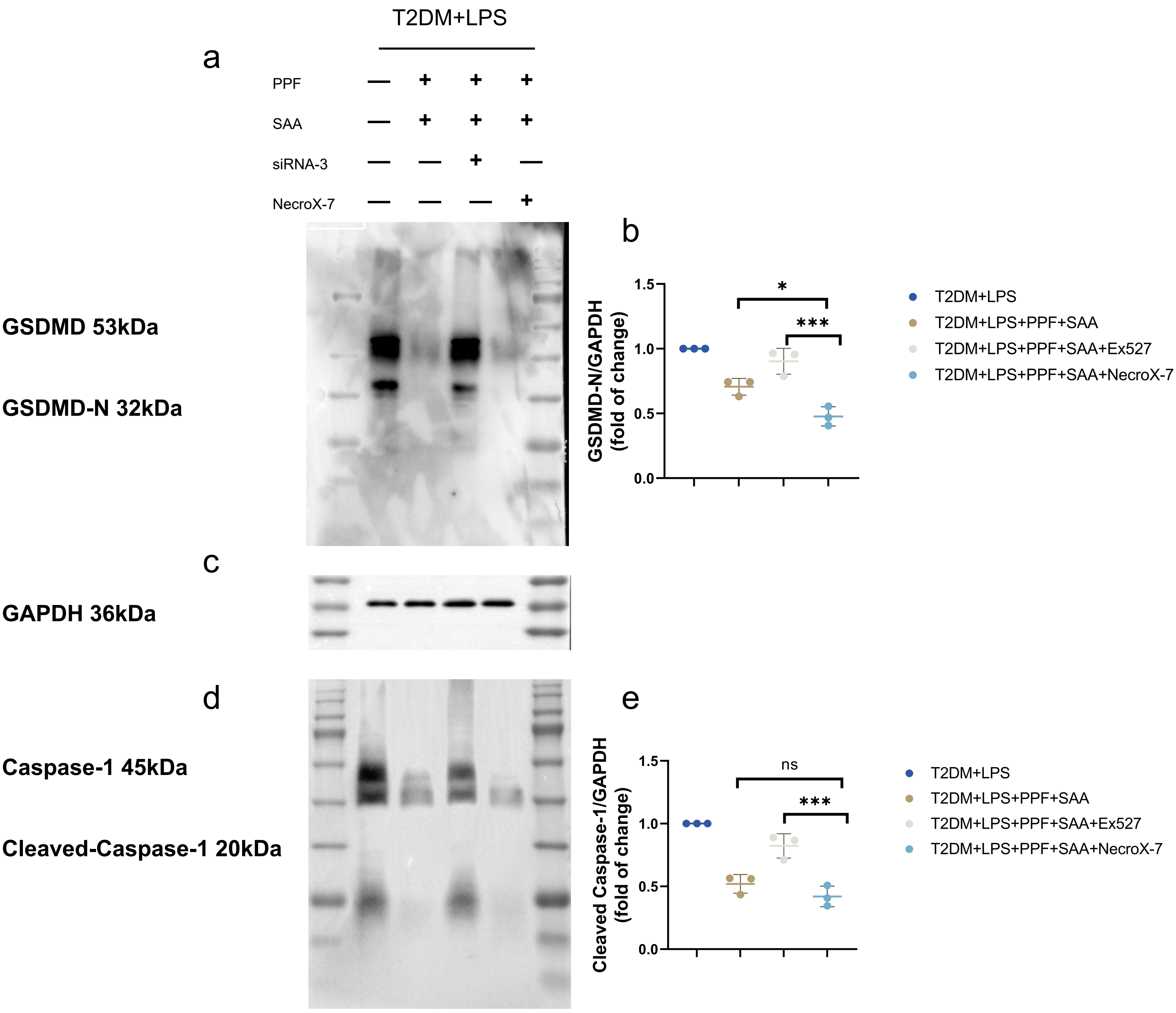

S6：Entire gels including full-length proteins as well as N-terminal or cleaved fragments in GSDMD and Caspase-1.

a) Representative image of western blot (GSDMD and GSDMD-N). b) GSDMD-N protein expression. c) Representative image of GAPDH. d) Representative image of western blot (Caspase-1 and Cleaved-Caspase-1). e) Cleaved-Caspase-1 protein expression. Data are expressed as the mean±SD, n=3 per group. $^{*}p < 0.05$, $^{**}p < 0.002$, $^{***}p < 0.001$, ns: not significant.
