# Supplementary material for: Propofol and Salvianolic Acid a Synergistically Attenuate LPS‐Induced Myocardial Pyroptosis in Diabetic Mice via the SIRT1/HMGB1 Pathway
Source: Mediators Inflamm. 2026 Jul 7;2026:6298056. doi: 10.1155/mi/6298056 (PMC13338769; doi:10.1155/mi/6298056)

Western blot in figure 2 for three repeats

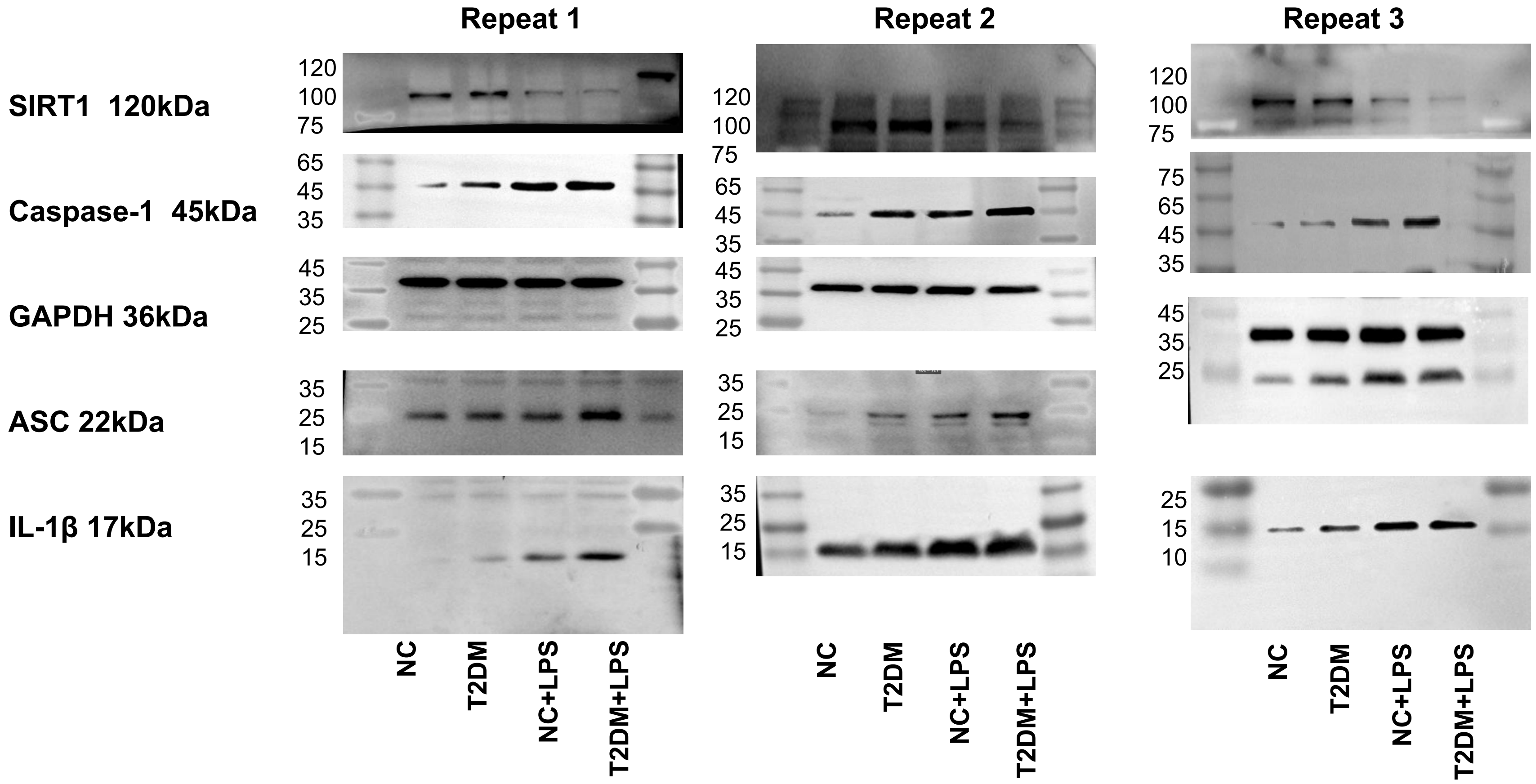

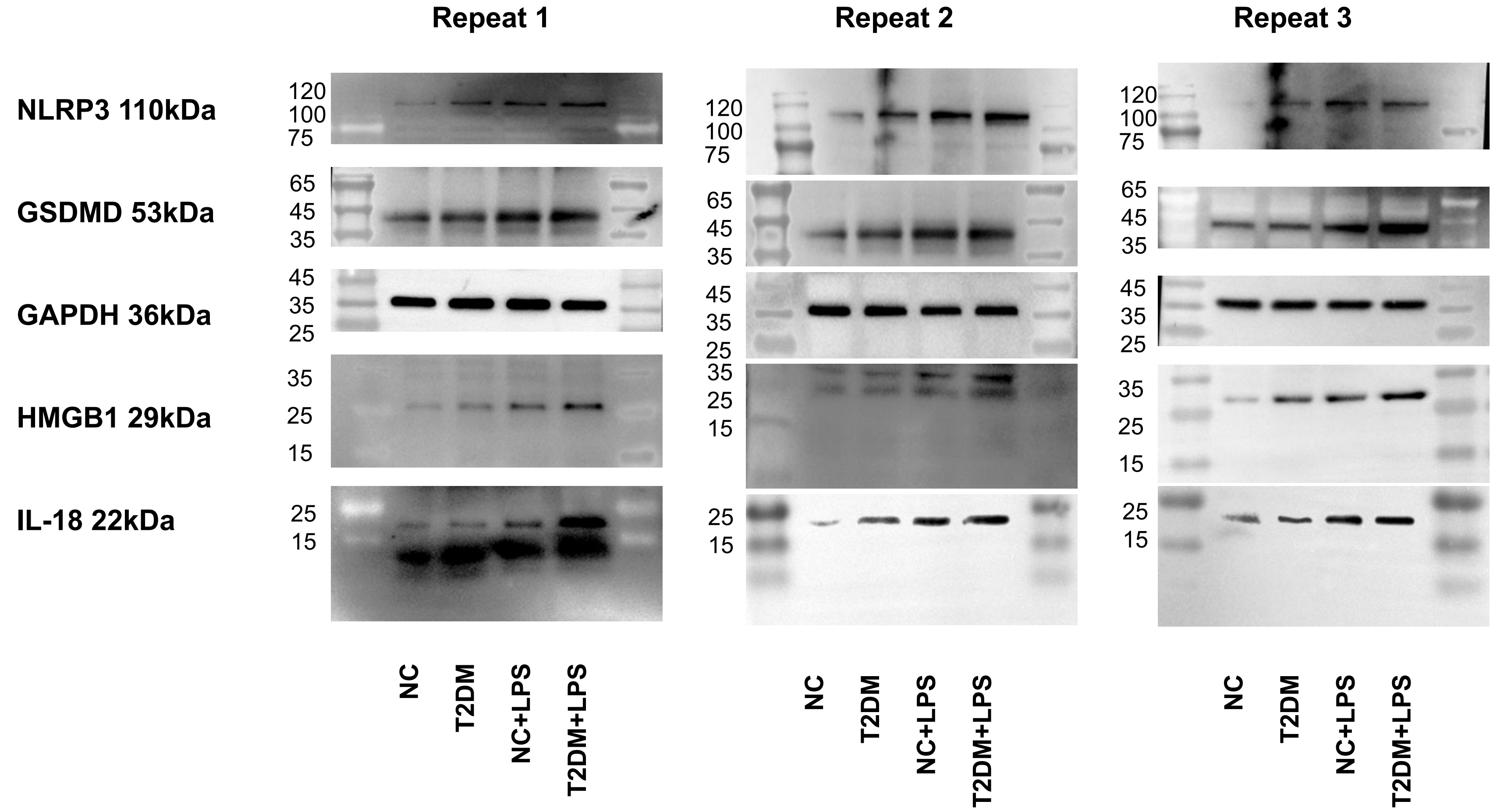

Western blot in figure 3 for three repeats

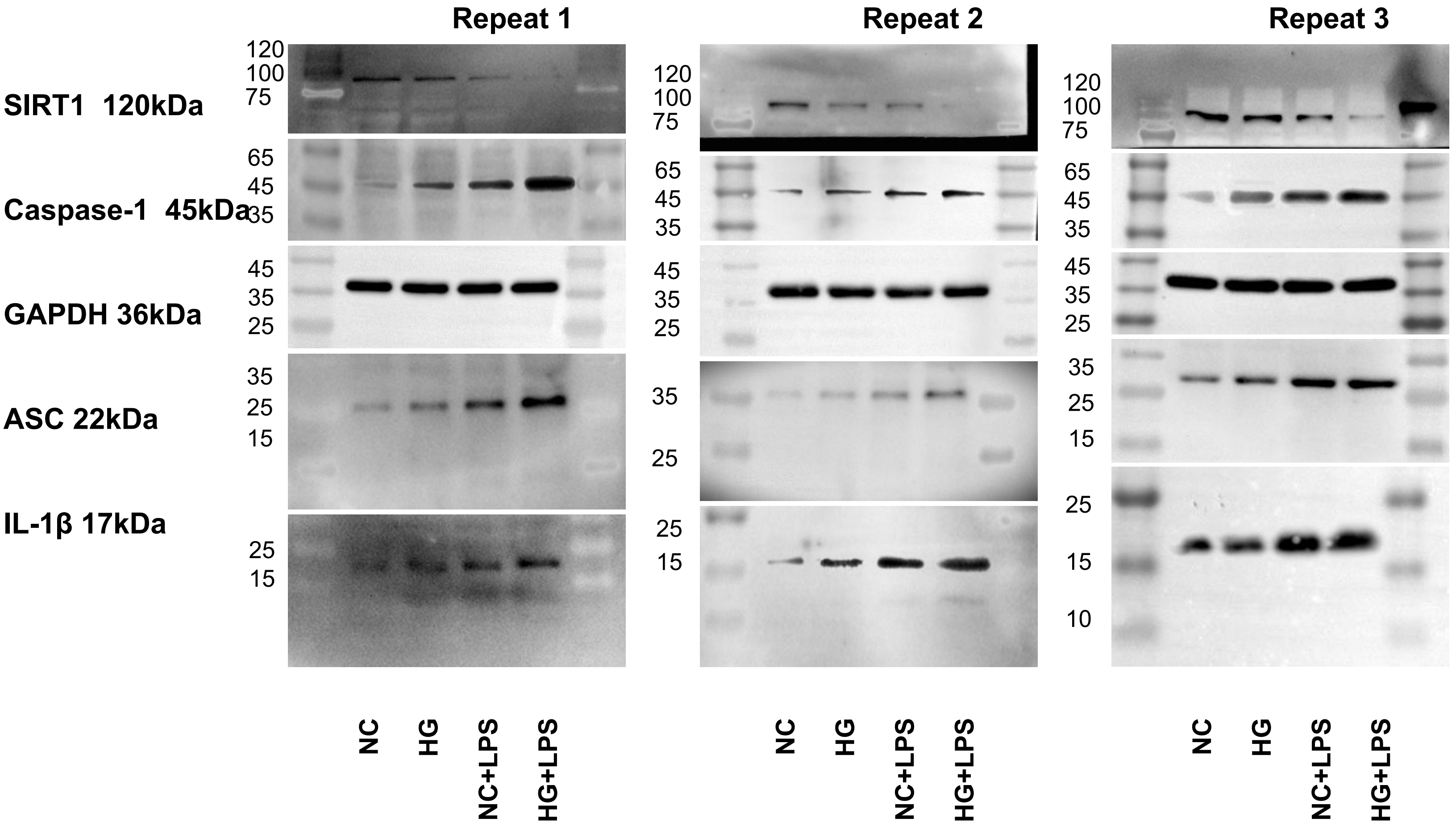

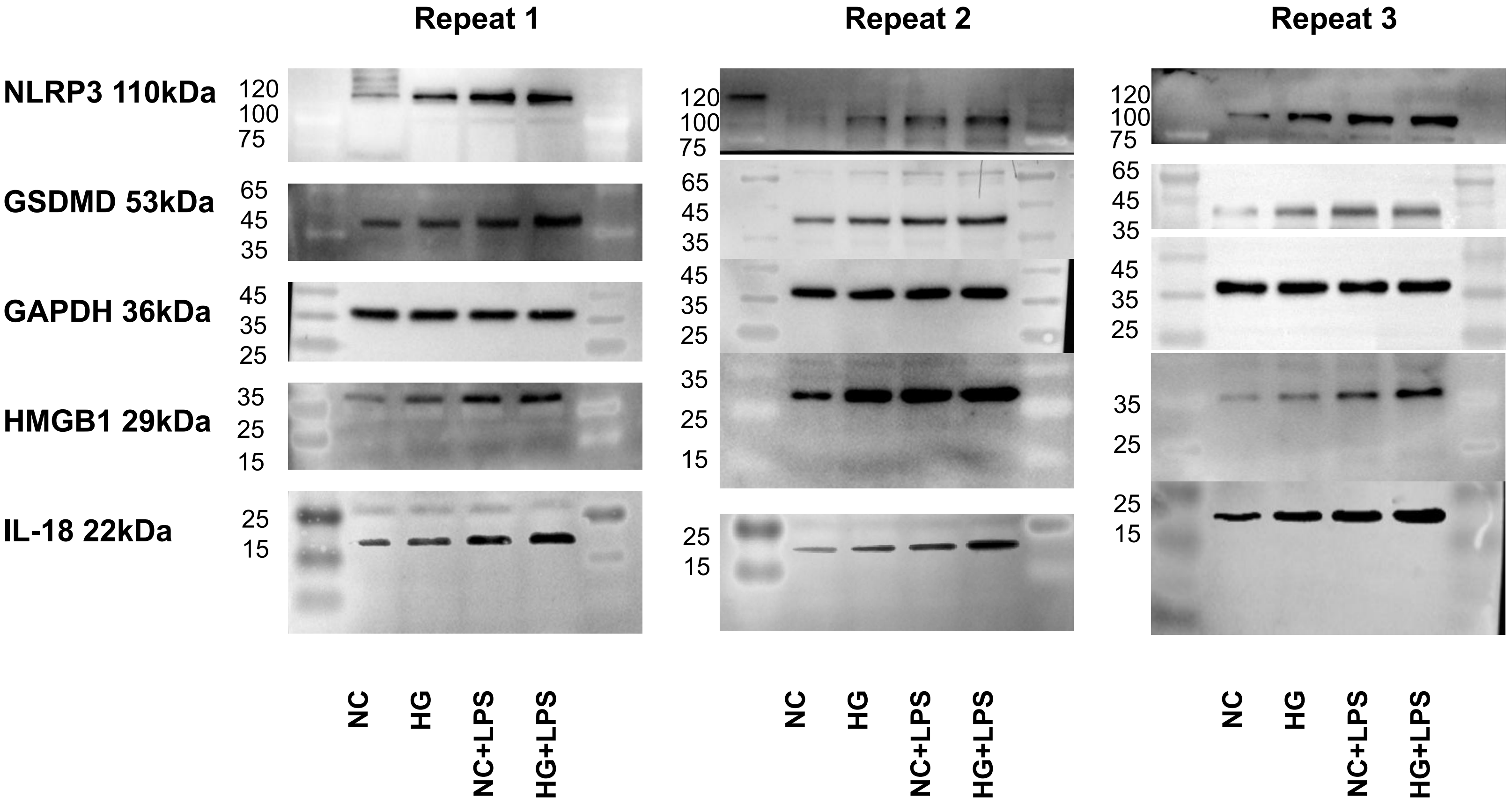

Western blot in figure 8 for three repeats

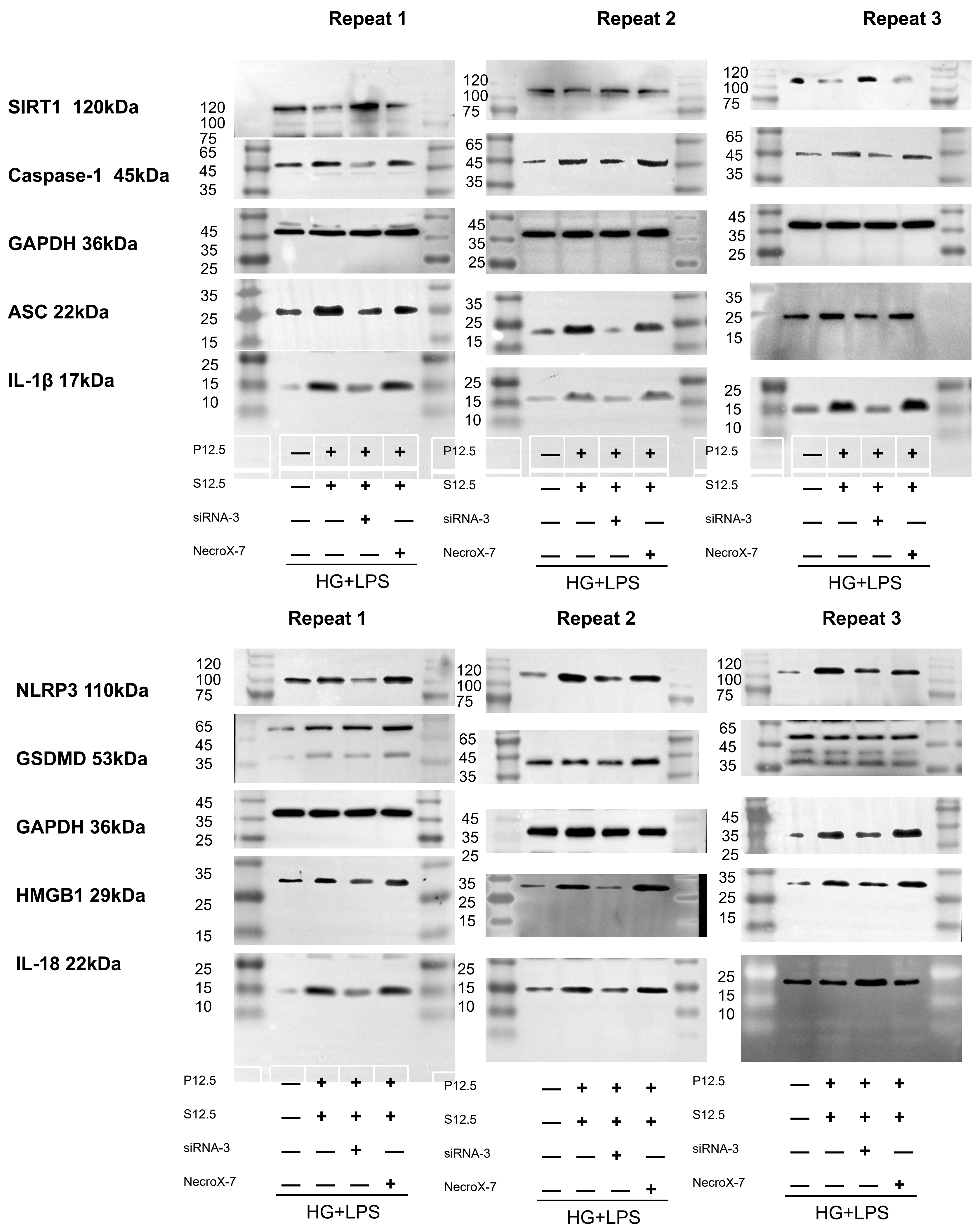

Western blot in figure 9 for three repeats

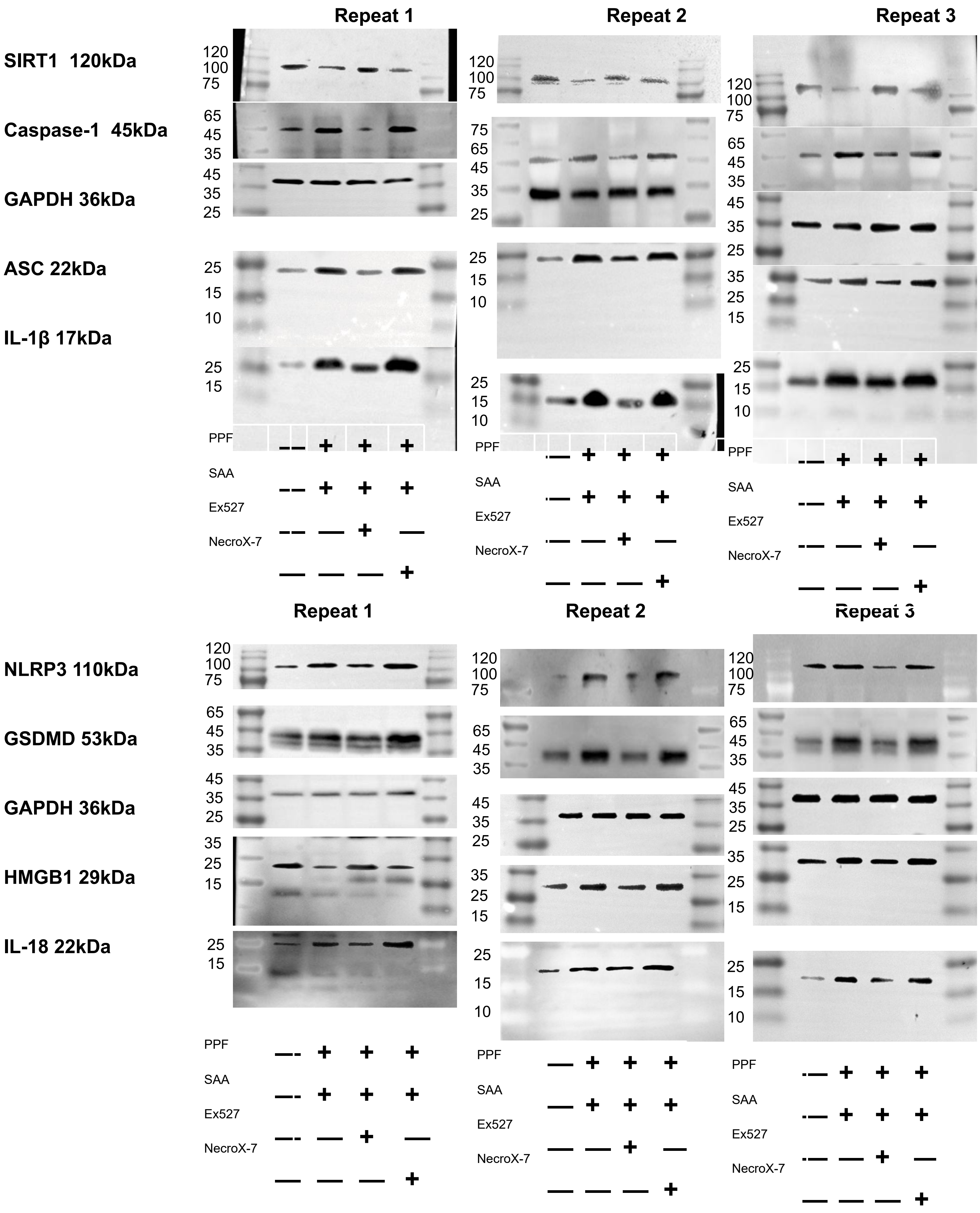

Western blot in figure 6 for three repeats

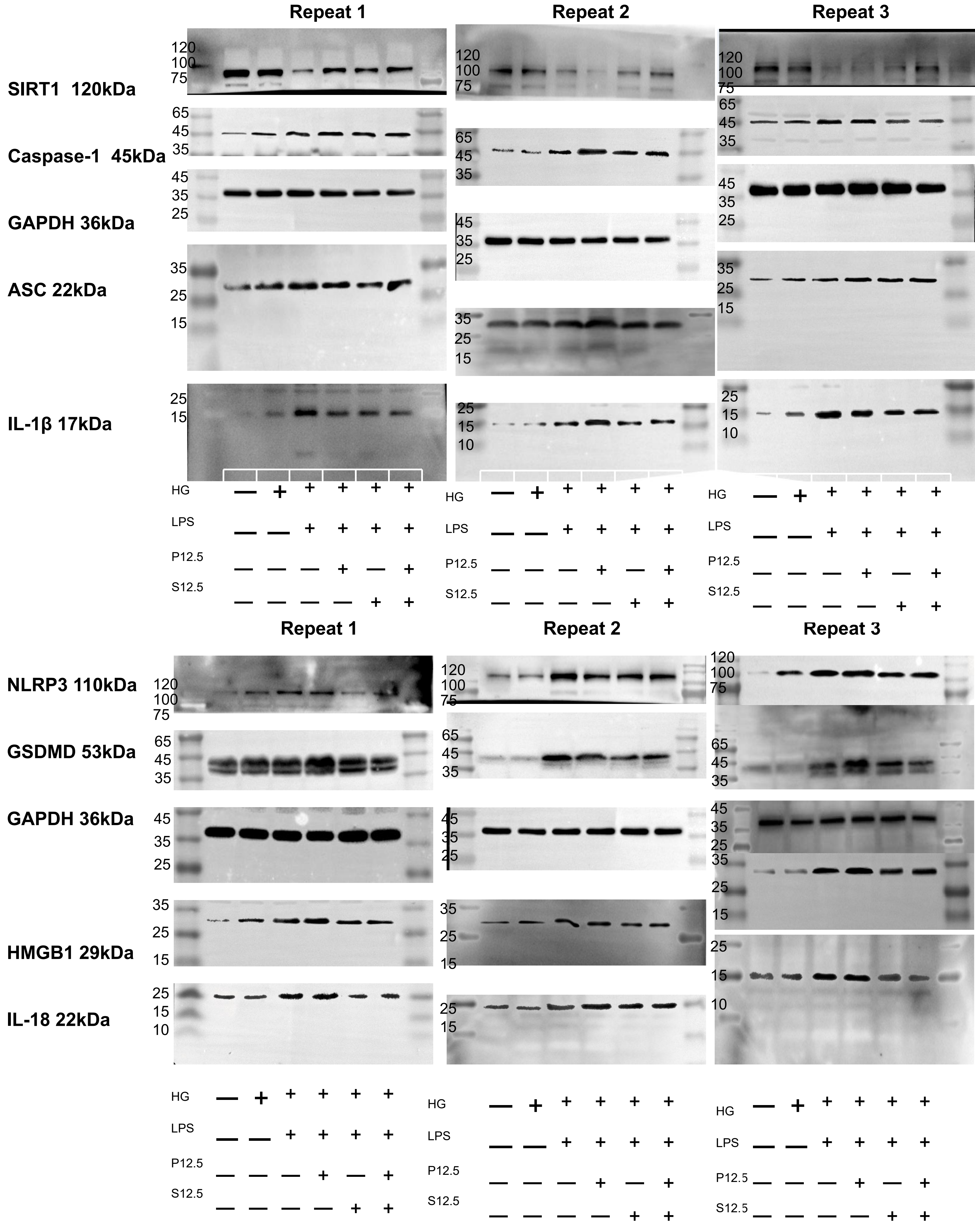

Western blot in figure 7 for three repeats

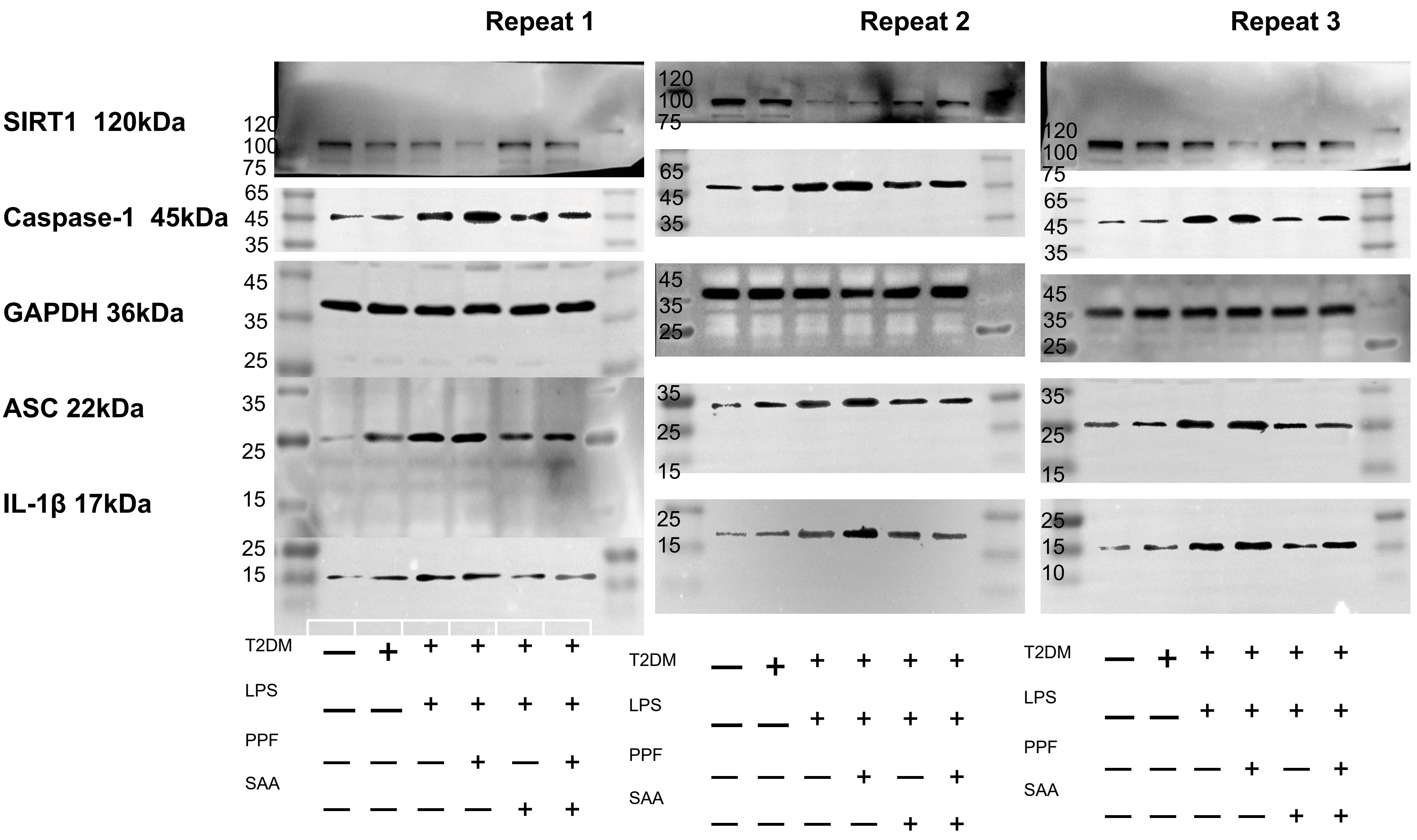

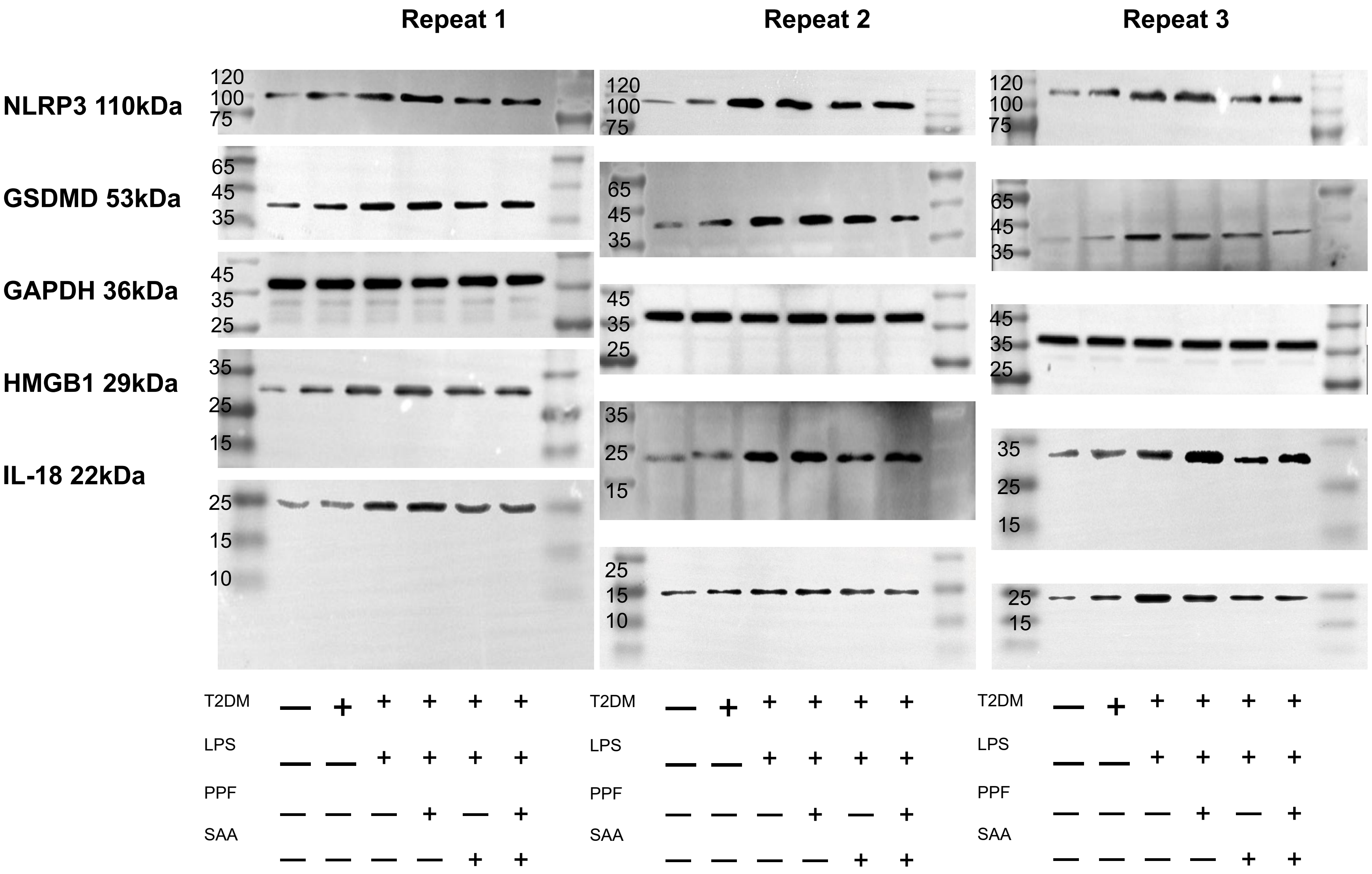

Supplement: Supplementary file 2 — Supporting Information 2 Complete Western blot gels showing all original replicates (n = 3) for the experiments described in this study. [file MI-2026-6298056-s002.pdf]
